# Advanced Age and Neurotrauma Diminish Glutathione and Impair Antioxidant Defense after Spinal Cord Injury

**DOI:** 10.1089/neu.2022.0010

**Published:** 2022-07-27

**Authors:** Andrew N. Stewart, Ethan P. Glaser, Caitlin A. Mott, William M. Bailey, Patrick G. Sullivan, Samir P. Patel, John C. Gensel

**Affiliations:** ^1^ Department of Physiology, University of Kentucky, Lexington, Kentucky, USA.; ^2^ Department of Spinal Cord and Brain Injury Research Center, University of Kentucky, Lexington, Kentucky, USA.; ^3^ Department of College of Medicine, University of Kentucky, Lexington, Kentucky, USA.; ^4^ Department of Neuroscience, University of Kentucky, Lexington, Kentucky, USA.

**Keywords:** aging, neuroprotection, neurotrauma, oxidative stress, Redox, sex differences

## Abstract

Advanced age at the time of spinal cord injury (SCI) exacerbates damage from reactive oxygen species (ROS). Mechanisms underlying this age-dependent response are not well understood and may arise from decreased antioxidant defense. We investigated how spinal cord levels of the antioxidant glutathione (GSH), and its regulation, change with age and SCI. GSH is used by GSH peroxidase to sequester ROS and is recycled by GSH reductase. Male and female, 4- and 14-month-old (MO) mice received a 60 kDyn contusion SCI, and the levels of GSH and its regulatory enzymes were evaluated at one and three days post-injury (dpi). The mice with SCI were treated with N-acetylcysteine-amide (NACA; 150 mg/kg), a cysteine supplement that increases GSH, to determine effects on functional and histological outcomes. GSH was decreased with older age in sham mice, and an SCI-dependent depletion was observed in 4-MO mice by three dpi. Neither age nor injury affected the abundance of proteins regulating GSH synthesis or recycling. GSH peroxidase activity, however, increased after SCI only in 4-MO mice. In contrast, GSH peroxidase activity was increased in 14-MO sham mice, indicating that spinal cords of older mice have an elevated oxidative state. Indeed, 14-MO sham mice had more oxidized protein (3-nitrotyrosine [3-NT]) within their spinal cords compared with 4-MO sham mice. Only 4-MO mice had significant injury-induced increases in 3-NT at three dpi. NACA treatment restored GSH and improved the redox environment in injured 4- and 14-MO mice at one dpi; however, three days of NACA delivery did not improve motor, sensory, or anatomical deficits at 28 dpi in 4-MO mice and trended toward toxicity in all outcomes in 14-MO mice. Our observation suggests that GSH levels at acute stages of SCI play a minimal role in age-dependent outcomes reported after SCI in mice. Collective results implicate elements of injury occurring after three dpi, such as inflammation, as key regulators of age-dependent effects.

## Introduction

Oxidative stress exacerbates tissue necrosis after spinal cord injury (SCI) and worsens functional outcomes.^1,2^ Reactive oxygen species (ROS) are derived acutely post-SCI from dysfunctional mitochondria^3,4^ and subacutely from microglia, macrophages, and neutrophils through nicotinamide adenine dinucleotide phosphate-oxidase enzyme activity (NOX).^**5–7**^ The pivotal role of ROS in exacerbating SCI lesions and worsening functional outcomes has been a focus of therapeutic investigations.^1,3,8,9^ The potential for antioxidant interventions to improve functional recovery after SCI highlights a need to better understand redox biology after neurotrauma.

Our laboratory previously identified that advanced age at the time of SCI increases oxidative stress and worsens functional outcomes in mice.^5,7,10,11^ Worse functional outcomes after SCI have also been reported in older clinical populations.^12^ This is concerning because the average age at time of SCI has increased from 27 to 42 years since the 1970s.^13^ While our previous studies have investigated age-associated sources of ROS production after SCI (mitochondria and macrophages),^7,11^ decreases in antioxidant defense with aging may also compound ROS damage.

Specifically, advanced age is associated with depleting available glutathione (GSH),^14–16^ an important regulator of antioxidant defense and free-radical sequestration. Age-dependent decreases in GSH have been reported in the brain^14–16^ but the role of age on GSH regulation in the spinal cord is understudied. Because SCI decreases available GSH in younger mice,^9,17^ we hypothesized that advanced age at time of SCI would compound GSH depletion after injury, thereby reducing antioxidant defenses and increasing oxidative damage in an age-dependent manner.

**FIG. 1.**
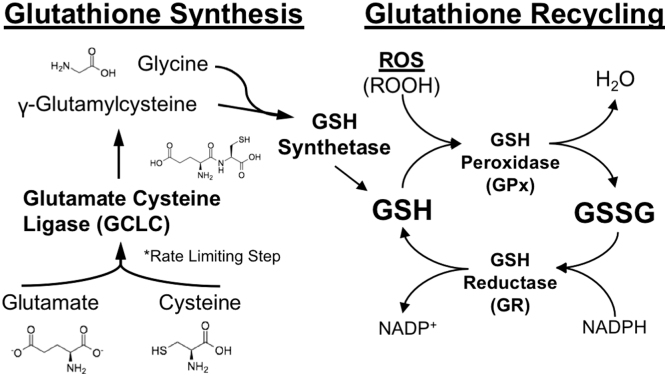
Glutathione synthesis and recycling. Glutathione is a recyclable tripeptide synthesized in two reactions. First, Glutamate-Cysteine Ligase ligates glutamate and cysteine, followed by glutathione synthetase, which adds glycine to finish the tripeptide structure. Two reduced glutathiones (GSH) are used as substrates for glutathione peroxidase (GPx) during the sequestration of reactive oxygen species (ROS). This oxidized glutathione (GSSG) is a dimer formed as a disulfide bridge that can be reduced by glutathione reductase (GR) back into two independent glutathione compounds using nicotinamide adenine dinucleotide phosphate (NADPH) as a substrate. GCLC, glutamate cysteine ligase catalytic domain.

Glutathione is a tripeptide synthesized in two ligation reactions.^18^ First, glutamate and cysteine are ligated at the catalytic domain of glutamate cysteine ligase (GCLC), which is considered the rate-limiting step to GSH synthesis. The resulting product, glutamylcysteine, is then ligated to glycine by glutathione synthetase (Fig. 1). Whether the rate-limiting step in GSH synthesis is enzymatic (GCL or GSH synthetase) or substrate dependent (glutamate, cysteine, or glycine) in nature is not well defined and is thought to be situationally dependent.^19,20^

Once produced, two GSH molecules sequester free radicals by reacting with various glutathione peroxidase (GPx) isoforms that display preferential affinity for different radical (i.e., ROS) substrates^21^ (Fig. 1). The resultant GSH molecules oxidize to form a disulfide (GSSG), which can be readily recycled back into usable GSH monomers by reduction with nicotinamide adenine dinucleotide phosphate (NADPH) and glutathione reductase (GR) (Fig. 1). This recycling system makes GSH a potent and reusable form of antioxidant defense at the expense of NADPH generated in the pentose-phosphate pathway.

One strategy to augment GSH with injury, pathology, or age is to increase substrate availability through cysteine supplementation using a variety of cysteine analogs.^9,17,22–26^ Supplementation using N-acetylcysteine (NAC) evokes promising neuroprotective results after both SCI and traumatic brain injury (TBI).^8,27,28^ The effects remain modest and inconsistent, however, which has been attributed to the low bioavailability of NAC and an inability to cross the blood–brain barrier.^29,30^

An analog to NAC, N-acetylcysteine amide (NACA), formed by replacing the hydroxyl with an amide group, confers greater blood–brain barrier and cellular membrane permeability.^29,30^ Treatment with NACA increases cellular GSH relative to NAC and is therefore a better cysteine supplement for disorders of the nervous system.^30^ Indeed, NACA treatment is consistently neuroprotective after SCI and TBI in young rats.^9,25,31,32^ Taken together, GSH dysfunction is evident after SCI, and strategies aimed at enhancing GSH have therapeutic potential.

This study aimed to identify how age compounds GSH dysfunction after SCI and determine whether the therapeutic effects of cysteine supplementation are age dependent. We hypothesized that aged (14-month-old [MO]) mice would present with more GSH dysfunction both before and after SCI, as well as experience a greater therapeutic benefit from NACA treatment. Our results only partially support this hypothesis.

We found that advanced age depletes GSH in mice in the absence of SCI; however, an injury-induced depletion of GSH was only consistently found in young (4-MO) mice. Most interestingly, while NACA did partially replenish GSH stores in both young and older mice after SCI, treatment with NACA did not improve outcomes in younger mice and trended toward toxic effects on all outcomes in older mice. Taken together, these data support an emerging understanding that older age at time of SCI can change both the pathophysiology and response to treatment. Our results raise concerns regarding the translation of pre-clinical findings in younger models of injury to the entire range of ages within the SCI demographic.

## Methods

### Animals, surgical procedures, and NACA delivery

All procedures were approved with the University of Kentucky's Institutional Animal Care and Use Committee. In total, data from *n* = 202 mice were used for these studies. All mice were group housed at 3–5 mice per cage under normal light cycle. The 14-MO (*n* = 95; originally from Jackson Laboratories and maintained at Charles River Laboratories by the National Institute for Aging) and 4-MO (*n* = 107; Jackson Laboratories) male and female C57/Bl6 mice were anesthetized using intraperitoneal (ip) injections of ketamine (100.0 mg/kg) and xylazine (10.0 mg/kg) and then received a surgical laminectomy followed by either 60 kDyn T9 contusion SCI (Infinite Horizons Impactor^33^) or laminectomy only (sham) as described previously.^5^

Mice received analgesic (Buprenex SR, 1.0 mg/kg), antibiotic (Enrofloxacin, 5.0 mg/kg), as well as saline (1.0 mL/day for up to 5 days) after surgery. Bladders from all SCI mice were manually expressed 2x/day for the duration of the study. In addition to the data obtained from 202 mice, four additional mice died during surgery and one other was euthanized for being moribund at 48 h post-SCI.

Mice of ages 4- and 14-MO were chosen as ages that represent two important benchmarks in clinical demographics. Specifically, 4-MO mice represent developmental stages corresponding to early adulthood in humans, which at 18–20 years old, is the most frequent age to receive an SCI.^13,34^ The 14-MO mice represent developmental stages corresponding to middle age, of which the average age at time of SCI in humans is 43 years of age.^13,34^ These ages were chosen to address concerns about the shift in demographics of SCI occurring at older ages since the 1970s and raise awareness about how modeling SCI in young rodents might not capture important physiological adaptations occurring during midlife aging. These age differences do not represent differences between young and elderly mice.

### Protein preparation

At 24- or 72-h post-injury, mice were again anesthetized with ketamine (4.0–5.0 mg) and xylazine (0.4-0.5 mg) and transcardially perfused with 0.1 M phosphate buffer (PB). A 6.0-mm section of spinal cord centered on the injury site was isolated and mechanically homogenized in 0.1 M PB containing protease inhibitors (Complete Mini, EDTA-FREE; 11836170001; Sigma Aldrich). Homogenates were sonicated on ice before addition of radioimmunoprecipitation assay (RIPA) buffer without sodium dodecyl sulfate (SDS; 20-188; Sigma Aldrich) and incubated for 20 min with gentle agitation.

Homogenates were centrifuged to pellet loose debris, and supernatants were allocated and frozen at -80°C or processed for Western blotting. The RIPA buffer was omitted when protein was isolated for GSH assessments to avoid lipidation of protein extract. Protein concentrations were assessed using bicistronic acid assay (BCA; 23225; ThermoFisher) and quantified against a standard curve prepared with bovine serum albumin (BSA).

### Western and dot blotting

Protein was prepared at 2.0 mg/mL in Laemmli buffer (1610747; Bio-Rad) with a final 2-mercaptoethanol (M3148; Sigma Aldrich) concentration of 5%. Protein was boiled for 5 min before loading 30 mg of protein into gels. Pre-made 4–20% gradient gels (4561096; Bio-Rad) were used, and protein was separated under reducing electrophoretic conditions. Protein was tank transferred in Tris/Glycine buffer (161073; Bio-Rad) containing 20% methanol onto nitrocellulose membranes (1620233; Bio-Rad). Membranes were transferred to blocking buffer containing 5% milk in Tris-buffered saline (TBS) for 30-min at room temperature.

Blots were imaged using fluorescent development (Odyssey CLx; LI-COR Biosciences) and probed sequentially against GR (56 kD; 1:2,000; ab124995; Abcam Co.) and GCLC (70 kD; 1:1,000; H00002729-M01; Novus Biologicals), followed by GPx-1 (22 kD; 1:1,000; VPA00488; Bio-Rad) and GSH Synthetase (56 kD; 1:2,000; VMA00364; Bio-Rad), and finally against GAPDH (36 kD; 1:5,000; ab9484; Abcam Co.). To account for having too many samples to fit onto a single blot, a sample of liver homogenate was loaded onto each gel and density differences between blots were used to normalize samples across blots.

3-nitrotyrosine (3-NT) or 4-hydroxy-nonenol (4-HNE) was assessed as a biomarker for free-radical damage generated downstream from ROS production.^2^ To assess 3-NT and 4-HNE, 2 μL of protein (1 mg/mL) was spotted onto a nitrocellulose membrane and allowed to dry overnight. Membranes were immersed in blocking buffer containing 5% BSA for 1 h at room temperature. Membranes were then incubated in antibodies derived against 3-NT (1:2,000; 06-284; Millipore Sigma) or 4-HNE (1:1,000; HNE11-S; Alpha Diagnostic International) overnight at room temperature followed by incubation in fluorescent conjugated secondaries (1:10,000; goat anti-IgG IRDye-680 or goat anti-IgG IRDye-800; LI-COR Biosciences) for 1 h before development.

For all Western blot and dot blot experiments *n* = 4–5 mice were used for each of the eight groups before combining sexes. When sexes were combined, *n* = 8–10 mice/group were used.

### GSH peroxidase and reductase assays

Protein allocations derived from spinal cord homogenate obtained at one and three dpi were used at 30-mg/well in duplicate. Commercial kits were obtained to analyze total GPx activity (ab102530; Abcam Co.), as well as total GR activity (ab83461; Abcam Co.). Baseline densities were obtained before starting the colorimetric assays, and kinetic readings were used to assess catalytic rates. Standard curves of obtained values were generated, and sample values were interpolated against best fit curves using Prism (version 9). For both activity assays at each time point, *n* = 4-5 mice were used per group before combining sexes. When sexes were combined, *n* = 8–10 mice/group were used.

### GSH determination

A housekeeping experiment was performed to determine at which time post-injury SCI exerted the greatest effects on GSH depletion. This first experiment utilized 4- and 14-MO, sham- and SCI-, female mice at both one and three dpi (*n* = 4–5) (Supplementary Fig. S1). Next, both sexes were included and 4- and 14-MO, male and female, sham and injured mice were used to determine effects of injury at three dpi (*n* = 4–5/group). Three dpi was chosen because it corresponded to the largest SCI-induced depletion in GSH from our first experiment.

The GSH content was assessed in tissue homogenate using deprotonated samples, with a starting concentration ranging from 0.67–1.2 mg protein/mL depending on the experiment. For each experiment, all samples were diluted to the same final protein concentration. Samples were pre-treated using 1 volume 5% 5-sulfosalicylic acid (SSA) to remove protein and tested using commercially prepared GSH colorimetric detection kits (EIAGSHC; ThermoFisher). To distinguish total GSH from free GSH, GSSG was tested in allocations of the same samples that were pre-treated with 2-vinylpyridine to alkylate and block free GSH from reacting. Reaction densities were obtained using kinetic readings and interpolated against standard curves using a four-parameter logarithmic best fit equation.

### GSH augmentation

To determine whether age or injury affect GSH synthesis capacity after SCI, naïve 4-MO female mice (*n* = 4) were treated with the cysteine analog NACA 1.5 h before tissue extraction (150 mg/kg/dose prepared at 150 mg/mL in saline; NACA was a gift from Sentient Lifesciences, New York, NY^9,25^). Protein was isolated as described above and diluted to 1 mg/mL for GSH analysis using colorimetric detection assays. For SCI animals, 4- and 14-MO female mice (*n* = 6–7/group) received SCI and were treated with 150 mg/kg NACA starting immediately post-injury, 12 h after SCI, and 24 h after SCI. Spinal cords were harvested 1.5 h after the last dose for GSH determination. Total and free GSH as well as GSSG and the GSH/GSSG ratios were determined.

### Cysteine substitution with NACA as a treatment for SCI

#### Treatment paradigm

To determine effects of cysteine replacement using NACA on long term outcomes after SCI, 4- and 14-MO female mice (*n* = 10) were given 60 kDyn SCI and treated immediately after injury with a booster injection of NACA (150 mg/kg) or saline. Next, primed osmotic pumps (1003D; Alzet Inc.) were implanted that were calibrated to deliver saline or 150 mg/kg/day of NACA for three days as published previously.^9^ At three days post-injury, mice were lightly anesthetized using isoflurane, and osmotic pumps were removed. Mice were examined for functional recovery for a total of 28 days after SCI. All behavioral and histological analyses were performed by experimenters who were blinded to group identities.

#### Locomotor assessments

Locomotor assessments were made using the horizontal ladder foot slip analyses^35^ and during open field exploration using the Basso Mouse Scale (BMS^36^). The BMS utilizes a 9-point rating scale to characterize gross locomotor functions ranging from complete paralysis (score 0) to normal functions (score 9) as mice explore an open field for 4 min. The BMS scores were obtained at one, three, seven, 14, 21, and 28 dpi by two experimental raters who were blinded to treatment groups. The horizontal ladder was used to determine the extent of sensory-motor coordination and was performed both pre-SCI, and at 28 dpi in mice capable of weight-supported stepping.

Video recordings were made of mice traversing the 1.0-m ladder, and events of foot slips below the ladder were counted. Foot slips were normalized to total steps taken by the respective hindlimb, and the average of both hindlimbs was taken for each trial. Three trials were used and averaged for each day of testing.

#### Thermal sensitivity

The Hargreave test of thermal hypersensitivity was used as described previously.^37,38^ Mice were acclimated to testing boxes for 2 h/day for two days before testing and similarly acclimated for at least 1 h before data acquisition. The Hargreave test was performed both pre-SCI as well as at 28 dpi. An infrared beam that was calibrated to reach 55°C over 25 sec was placed under the plantar surface of the hindpaws of mice as they were standing still. A deliberate flinch, indicated by an abrupt lifting and replacement of the paw, or picking up and licking of the paw, was counted as an intentional movement, and the duration of stimulation before the intentional movement was recorded.

Trials where mice began walking without an explicit reaction to the heat were not considered valid and were discarded. The paws were given at least 2 min of rest or longer before the next trial. In total, at least three trials were obtained per foot, and the obtained numbers were averaged for both feet to generate one score per subject per time point.

### Histology and immunohistochemistry

At 28 dpi, mice were anesthetized using an overdose of ketamine (4.0–5.0 mg) and xylazine (0.4–0.5 mg) and euthanized via transcardial perfusion using phosphate-buffered saline (PBS) followed by 4% formaldehyde made from paraformaldehyde prills (Millipore Sigma). Spinal cords were extracted and post-fixed in 4% formaldehyde for 2 h at room temperature before being transferred to 0.1 M PB overnight. Spinal cords were acclimated to 30% sucrose for dehydration, embedded in Optimal Cutting Temperature-Compound (OCT), and frozen on dry ice. Tissue was blocked together with at least one cord from each group randomly selected and placed per block. Blocks were sectioned coronal at 10 μm thickness with every 10th section being collected per slide.

Tissue sparing and neuron survival were assessed in sequential sets of tissue. Tissue sparing was assessed by immunolabeling against neurofilament-200 kD (1:1,500: Ck x NF200; NFH; Aves Labs) and counterstained using eriochrome cyanine to evaluate white matter as described previously.^11^ Neuron survival was assessed by immunolabeling against neuronal-specific nuclei (1:4,000; Rb x NueN; NBP1-77686; Novus Biologicals). Primary antibodies were targeted using biotinylated secondary antibodies against the host species (1:500, goat anti-chicken, BA9010, or goat anti-rabbit, BA-1000; Vector Laboratories) followed by incubation using avidin biotin complex (ABC; 1:200; PK-6100; Vector Laboratories) and developed using 3,3'-diaminobenzidine (DAB) with or without nickel.

All sections received antigen retrieval at 80°C in sodium citrate buffer with 0.1% Tween-20 (pH 6.0) for 5 min, were then treated with 0.3% H_2_O_2_ in 40% methanol in PBS to quench endogenous peroxidase activity for 30 min, then incubated in 5% normal goat serum in PBS/0.1% Triton-X 100 for 1 h at room temperature before primary antibody incubation overnight at room temperature. Stained slides were dehydrated using graded ethanol dilutions, cleared using Histoclear (101412-878; VWR Scientific), and coverslipped using Permount (SP15-500; Fisher Scientific). Slides were imaged using Axioscan (model Z1, Carl Zeiss AG., Oberkochen, GE) at 20x magnification and visualized and quantified using Halo software (Indica Labs, Albuquerque, NM).

To assess tissue sparing, the white matter and intact gray matter were traced in each section within 700 mm rostral and caudal to the lesion epicenter. The lesion epicenter was objectively determined as the section containing the least amount of spared tissue. All analyzed sections were oriented with respect to this objectively defined epicenter. To account for size differences between 4- and 14-MO spinal cords, the total spared tissue was divided by the total tissue area to get a percent spared tissue.

To analyze spared neurons, a cell-counting algorithm was created on Halo and applied to all stained sections as described previously.^11^ The cell-counting algorithm allows for distinguishing cells based on size, morphology, and staining density and applies the standards consistently across all sections in all samples. Neurons in the ventral horns were counted by limiting the analysis view to all neurons below the central canal. Every section up to 700 mm rostral and caudal to the lesion epicenter was counted.

### Statistics

Three-way analysis of variance (ANOVA) was performed for Western blots, dot blots, activity assays, and GSH assays to determine main effects of injury, sex, and age as well as to screen for significant interactions. If there were no significant sex-by-age or sex-by-injury interactions or main effects of sex, data from both sexes were collapsed, and two-way ANOVAs were performed against injury and age. If sex effects were found, data from both ages were collapsed, and a two-way ANOVA was performed against injury and sex. When significant sex-by-age interactions were detected, pair-wise comparisons against all groups were performed to evaluate for injury effects within a group. Otherwise, pair-wise comparisons were made across collapsed groups.

For GSH determination after NACA treatment, only female mice of ages 4- and 14-MO were used, which was assessed using two-way ANOVA. Mice in NACA treated experiments were performed in cohorts of a single age, so data were either normalized to the average of vehicle-treated mice to control for batch-to-batch variability or assessments were limited within a single age. Two-way ANOVA was used to compare between-group effects in horizontal ladder, Hargreave test, tissue sparing at the lesion epicenter, and neuron survival.

Two-way repeated-measures ANOVA was used to evaluate total tissue sparing throughout the length of the analyzed lesion (distance x group), and individual two-way ANOVAs were performed to assess effects of NACA on BMS scores and BMS subscores (time x group). The Sidak pairwise comparisons were used as a *post hoc* test when appropriate. Significant α levels were set to less than or equal to *p* values of 0.05.

## Results

### Decreased GSH with age and SCI corresponds with an antioxidant transcriptional profile

#### Age and SCI independently decrease spinal levels of GSH and increase oxidative stress

**FIG. 2.**
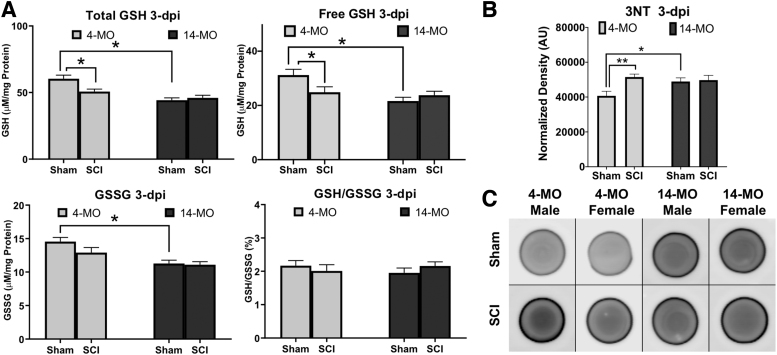
Age and spinal cord injury (SCI) diminish spinal glutathione and increase oxidative stress. (**A**) Both total and free reduced glutathione (GSH) are diminished in 4-month-old (MO) mice three days after SCI, while total, free, and oxidized GSH are diminished in 14-MO sham-injured mice. (**B**) 3-Nitrotyrosine (3-NT) as a measure of oxidative stress was evaluated using dot blot (**C**) and was increased in 4-MO mice after SCI, as well as increased in 14-MO, relative to 4-MO, sham-injured mice. Dot densities are representative of group means. All assessments were performed using two-way analysis of variance with Sidak pair-wise comparisons as *post hoc*. *n* = 10/group. Graphs represent mean ± standard error of the mean. *p < 0.05, ** *p* < 0.01. dpi, days post-injury; GSSG, oxidized glutathione.

First, we sought to determine the relationship between age and SCI on spinal cord GSH levels. Based on an initial experiment in a single sex (females), we found an injury effect in both 4- and 14-MO animals at three dpi (Supplementary Fig. 1) and chose this time point for detailed analyses. We examined total GSH (combined free and oxidized form), GSH in its reduced (free GSH) and oxidized (GSSG) forms, as well as the GSH/GSSG redox ratio in sham and SCI animals using both male and female, 4- and 14-MO, mice (Fig. 2).

Three-way ANOVA did not reveal a significant effect of sex on total GSH levels after SCI (F_(1,32)_ = 3.56, *p* = 0.068) or free GSH (F_(1,32)_ = 0.62, *p* = 0.24) so sexes were combined. Two-way ANOVA produced both a main effect of age on total- (F_(1,36)_ = 24.19, *p* < 0.001) and free-GSH (F_(1,36)_ = 9.15, *p* < 0.01), as well as injury-by-age interactions (total, F_(1,36)_ = 7.21, *p* < 0.01; free, F_(1,36)_ = 5.72, *p* < 0.05). Specifically, GSH was significantly lower in the sham-injured spinal cords of 14- compared with 4-MO sham-mice (total, *p* < 0.001; free, *p* < 0.001). An injury-effect was only observed in 4-MO mice for total (*p* < 0.01) and free GSH (*p* < 0.05; Fig. 2A). These findings indicate that advanced age at time of SCI pre-dispose the spinal cord to oxidative stress by having less available GSH to sequester ROS at the time of injury.

Previous work from our laboratory has identified elevated levels of ROS damage in the injured spinal cords of 14-MO versus 4-MO mice by seven dpi^5,11^ but not at three dpi.^5^ Our previous work, however, did not compare ROS accrual compared with sham-injured mice. Here we found that 3-NT levels in isolated proteins paralleled findings of GSH. Specifically, no sex effect was observed using a three-way ANOVA (F_(1,31)_ = 0.01, *p* = 0.91), but both a significant injury effect (F_(1,35)_ = 6.31, *p* < 0.05) and age-by-injury interaction (F_(1,35)_ = 4.67, *p* < 0.05) was found when combining sexes.

3-NT was significantly increased in sham-injured 14- compared with 4-MO mice (*p* < 0.05), which is consistent with a decrease in GSH found with advanced age. Similarly, only 4-MO mice had a significant injury-induced increase in 3-NT at three dpi (*p* < 0.01; Fig. 2B,C). Not observing an increase in oxidative stress between 4- and 14-MO SCI-mice at three dpi is consistent with our previous reports.^5,11^

#### GCLC and GSH synthetase expression are unaffected by age and SCI

**FIG. 3.**
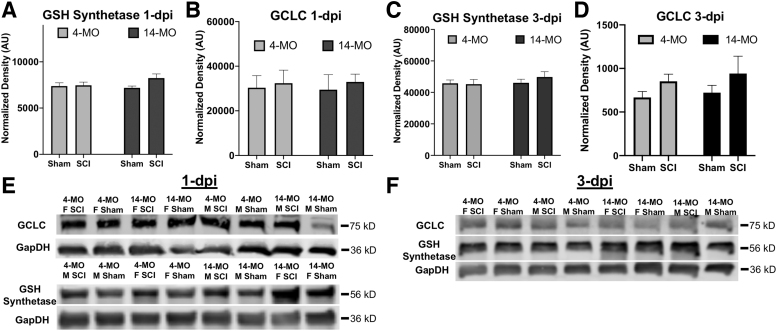
The glutathione synthesis enzymes, GCLC and GSH synthetase, are not affected by age or spinal cord injury (SCI). Both enzymes involved in GSH synthesis were evaluated using Western blots (**A–F**) to determine how age and injury effect protein expression. (**A,C**) The GSH synthetase was not affected by age, sex, or SCI at either one or three days post-injury (dpi). (**B,D**) GCLC expression was not affected by age, sex, or SCI at either one or three dpi. Assessments were performed using two-way analysis of variance. *n* = 9–10/group. Graphs represent mean ± standard error of the mean. *p < 0.05, **p < 0.01. GCLC, glutamate cysteine ligase catalytic domain; GSH, reduced glutathione.

We next sought to determine whether age and SCI affect the pathways that synthesize and regulate GSH. Western blots were used to compare the relative abundance of two enzymes that synthesize GSH—specifically, GSH synthetase and GCLC, of which GCLC is the first and rate limiting step to GSH synthesis (Fig. 1). Protein expression was examined in sham, one, and three dpi conditions in 4- and 14-MO, male and female, mice. Both GSH synthetase and GCLC were stably expressed across both ages, both sexes, and all injury time points (Fig. 3A,B).

Specifically, for GSH synthetase, no differences were found for sex (one dpi, F_(1, 32)_ = 0.535, *p* = 0.47; three dpi, F_(1, 32)_ = 0.268, *p* = 0.60), age (one dpi, F_(1, 32)_ = 0.739, *p* = 0.39; three dpi, F_(1, 32)_ = 0.747, *p* = 0.39), or injury (one dpi, F_(1, 32)_ = 2.77, *p* = 0.10; three dpi, F_(1, 32)_ = 0.306, *p* = 0.58). Similarly, no differences were found in GCLC abundance across sex (one dpi, F_(1, 31)_ = 0.872, *p* = 0.35; three dpi, F_(1, 32)_ = 1.927, *p* = 0.17), age (one dpi, F_(1, 31)_ = 0.007, *p* = 0.93; three dpi, F_(1, 32)_ = 0.369, *p* = 0.54), or injury (one dpi, F_(1, 31)_ = 0.30, *p* = 0.57; three dpi, F_(1, 32)_ = 2.917, *p* = 0.097) (Fig. 3F). Collectively, these data suggest that neither age nor SCI change the expression of the two enzymes responsible for GSH synthesis.

#### Age and SCI independently increase the activity of GSH peroxidase and reductase

**FIG. 4.**
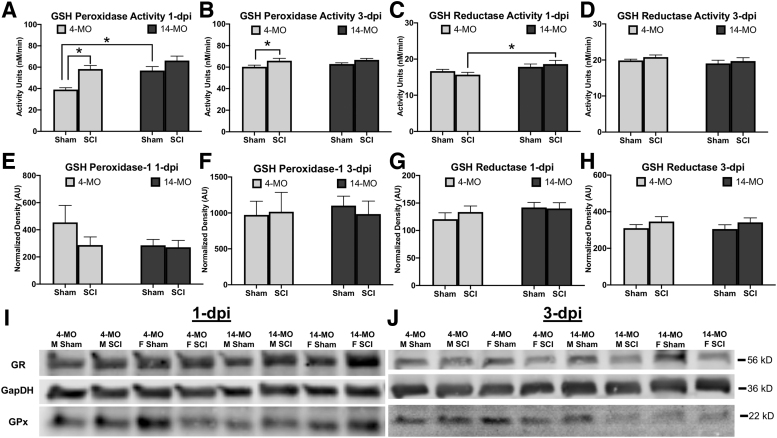
Glutathione peroxidase (GPx) activity is increased after spinal cord injury (SCI). Two enzymes that use and recycle reduced glutathione (GSH) to sequester reactive oxygen species (ROS) were evaluated using activity assays (**A–D**) and Western blots (**E–J**) to determine how age and injury effect protein function and expression. (**A,B**) The GPx activity is significantly increased with age between sham-mice at one day post-injury (dpi) but only significantly increased after SCI in 4-month-old (MO) mice at one and here dpi. (**C,D**) Glutathione reductase (GR) was not increased after SCI relative to sham controls but was higher in 14- compared with 4-MO mice after SCI. (**E–H**) While increases in enzyme activity were found for GPx and GR, no differences were found at the level of protein expression at either day. Assessments were performed using two-way analysis of variance with Sidak pair-wise comparisons as *post hoc*. *n* = 9–10/group. Graphs represent mean ± standard error of the mean. *p < 0.05.

We next evaluated the effects of age and SCI on the protein expression, and activity, of GPx-1 and GR—the enzymes that use and recycle cellular GSH. Western blots were used to identify sex, age, or injury effects in sham-, one, and three dpi samples and did not detect any differences in protein expression between groups for GPx-1 or GR (Fig 4.C,D). Activity assays, however, for total GPx at one dpi did reveal a significant main effect of injury (F_(1, 32)_ = 21.21, *p* < 0.0001), age (F_(1, 32)_ = 17.32, *p* < 0.001), and sex (F_(1, 32)_ = 5.43, *p* < 0.05) with no significant interactions (Fig. 4A).

Pairwise comparisons after two-way ANOVA against injury and age revealed a significant injury effect in 4- (*p* < 0.001), but not 14-MO mice (*p* = 0.113), with significant increases in 14- compared with 4-MO sham-injured mice (*p* < 0.01). When combining ages, pairwise comparisons revealed a significant injury effect in both male (*p* < 0.05) and female mice (*p* < 0.05), but no differences between male and female sham (*p* = 0.33) or SCI conditions (*p* = 0.37). At three dpi, three-way ANOVA revealed a significant effect of injury (F_(1, 30)_ = 8.55, *p* < 0.01) that was detected in 4- (*p* < 0.05), but not 14-MO mice (*p* = 0.18) after combining sexes (Fig. 4A).

In contrast to a robust injury effect detected for GPx activity, no injury effect was detected at one dpi for GR (F_(1, 32)_ = 0.02, *p* = 0.88). There is, however, a significant effect of age (F_(1, 32)_ = 8.33, *p* < 0.01) and sex (F_(1, 32)_ = 4.5, *p* < 0.05), with significantly greater GR activity in 14- compared with 4-MO mice at one dpi (*p* < 0.05), but not sham injuries (*p* = 0.48) (Fig. 4B). No effects were found for GR activity at three dpi. Despite not identifying differences in the protein abundance of GPx-1 or GR in Western blot, total activity of GPx was increased in an age- and SCI-dependent manner, while GR was increased in an age-dependent manner at one dpi. These data reveal an interaction that suggests either other GPx isoforms are upregulated with injury or post-translational modifications are increasing the catalytic rate of the available enzymes after SCI.

### Cysteine supplementation partially restores spinal GSH in both 4- and 14-MO mice after SCI

An apparent contradiction in our data is that total and free GSH levels are diminished with age and SCI despite no decrease in GCLC, the rate limiting enzyme in GSH synthesis. This suggests that the rate limiting step to GSH synthesis is likely because of substrate availability rather than limited availability of the enzyme. We tested to see whether increasing available cysteine would augment GSH *in vivo* by delivering the cysteine analog, NACA.

**FIG. 5.**
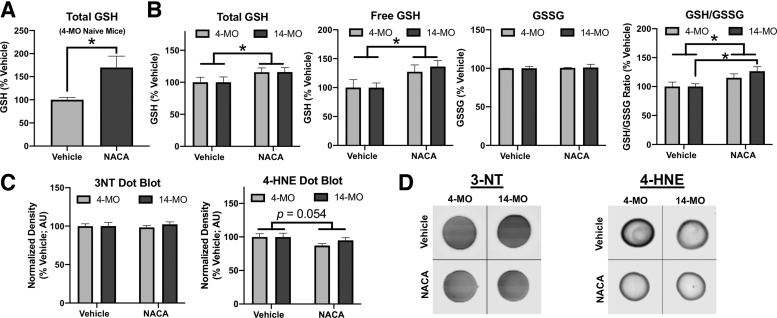
Cysteine supplementation using N-acetylcysteine amide (NACA) increases spinal levels of reduced glutathione (GSH) and improves the redox environment after SCI. (**A**) NACA (150 mg/kg) was given to naïve female mice to determine the efficacy of increasing spinal levels of GSH at 1.5 h post-treatment, which significantly elevated levels to approximately 170% of vehicle-treated controls. (**B**) When NACA (150 mg/kg/day) was delivered to mice for 24 h after spinal cord injury (SCI), both total and free GSH as well as the redox ratio (GSH/GSSG) are increased relative to vehicle-treated controls. (**C,D**) Dot blots performed against 3-nitrotyrosine (3-NT) and 4-hydroxy-nonenol (4-HNE) were used as measures of oxidative stress accumulation and revealed no significant effects of NACA on 3-NT or 4-HNE. (A) Assessment using two-tailed *t* test. (B,C) Assessments were performed using two-way analysis of variance with Sidak pair-wise comparisons as *post-hoc*. (A) *n* = 4/group (B,C) *n* = 6–7/group. Graphs represent mean ± standard error of the mean. *p < 0.05.

First, we treated a small cohort of 4-MO naïve female mice to reproduce previous literature that demonstrates NACA can increase GSH within the central nervous system.^30^ Indeed, NACA treatment at 150 mg/kg increased spinal GSH levels (*p* < 0.05) to approximately 170% of untreated controls, validating NACA's ability to cross the intact blood–brain barrier and augment total spinal GSH (Fig. 5A). Next, mice with SCI were treated with either saline as a vehicle control or 150 mg/kg NACA for 24 h post-injury before assessing GSH. Independent of age and compared with vehicle-treated mice, treatment with NACA significantly increased total (F_(1, 24)_ = 4.59, *p* < 0.05) and free spinal GSH levels (F_(1, 22)_ = 7.71, *p* < 0.01).

Further, NACA improved the GSH/GSSG redox ratio at 24 h post-injury when ages were combined (F_(1, 22)_ = 9.59, *p* < 0.01), as well as specifically in 14-MO mice (*p* < 0.05). Observing a similar increase in GSH levels after NACA treatment between ages is consistent with finding similar levels of GCLC within the spinal cords of 4- and 14-MO mice and validates that available cysteine is a substrate limitation to GSH production.

To determine whether NACA mitigated oxidative stress, we performed dot blots to assess the relative abundance of 3-NT and 4-HNE accumulation in NACA-treated mice at 24 h post-SCI. Dots from NACA-treated mice displayed trends toward a main effect of NACA on reducing 4-HNE (F_(1, 24)_ = 4.10, *p* = 0.054), but not in 3-NT (F_(1, 24)_ = 0.01, *p* = 0.91; Fig. 5C,D). In conjunction with the above amassed data, these findings indicate that available cysteine is the rate limiting reaction to GSH synthesis with age and SCI and that administering cysteine supplements partially restores spinal levels of GSH and improves the redox environment.

### Delivery of NACA to treat SCI does not influence recovery in 4- or 14-MO mice

#### Motor abilities, sensory functions, and tissue pathology were not significantly affected by NACA treatment

To determine whether NACA treatment and associated reductions in oxidative stress can exert long-term effects on SCI outcomes, we treated SCI mice with NACA for 72 h post-SCI as described previously.^9^ Briefly, a booster dose of NACA (150 mg/kg) was delivered to mice immediately after SCI, and primed osmotic pumps were implanted to deliver NACA at a calibrated rate of 150 mg/kg per day. Motor recovery was assessed using the BMS scale of locomotor recovery as well as horizontal ladder, and thermal hypersensitivity was determined using the Hargreave test.

**FIG. 6.**
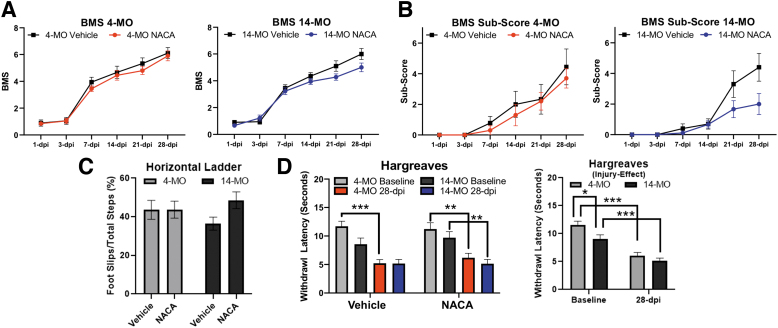
Treatment with N-acetylcysteine amide (NACA) does not improve locomotor or sensory outcomes after spinal cord injury (SCI). (**A-C**) NACA (150 mg/kg/day) was given to 4- and 14-MO mice after SCI and locomotor outcomes were assessed on the Basso Mouse Scale (BMS) (**A**) and BMS subscore (**B**), and horizontal ladder at 28 days post-injury (dpi) (**C**). No significant effects were observed between groups on any outcome. (**D**) Thermal hypersensitivity was evaluated using the Hargreaves testonce before and once at 28 days after SCI. No effects were found after NACA treatment, but significant hypersensitivity was observed after injury in both groups, with older mice having more hypersensitivity before SCI. (A,B) Assessments were performed using two-way repeated measures analysis of variance (ANOVA) for each age separately. (C) Assessment were performed using two-way ANOVA. (D) Assessment were performed using three-way ANOVA before combining treatments, which followed using a two-way ANOVA; Sidak pair-wise comparisons were used as *post hoc*. *n* = 9-10/group. Graphs represent mean ± standard error of the mean. *p < 0.05, **p < 0.01, ***p < 0.001.

After 28 days of recovery, NACA treatment did not affect BMS (F_(1, 17)_ = 0.43, *p* = 0.51; Fig. 6A), BMS subscores (F_(1, 17)_ = 0.24, *p* = 0.63; Fig. 6B), or horizontal ladder foot slips in 4-MO mice (*p* = 0.99; Fig. 6C). Similarly, there was no effect of NACA treatment on SCI recovery in 14-MO mice although there were trends toward a toxic effect: BMS (F_(1, 17)_ = 3.46, *p* = 0.08; Fig. 6A), BMS subscore (F_(1, 17)_ = 3.69, *p* = 0.07; Fig. 6B), and horizontal ladder foot slips in 14-MO mice (*p* = 0.10; Fig 6C). Three-way repeated-measures ANOVA evaluating thermal hypersensitivity did not reveal any effects of NACA (F_(1, 34)_ = 0.34, *p* = 0.56) but did reveal both a significant effect of injury (F_(1, 34)_ = 66.04, *p* < 0.0001) and age (F_(1, 34)_ = 4.53, *p* < 0.05), with older mice having more sensitivity to heat before SCI (*p* < 0.01) after combining treatments within an age (Fig. 6D).

**FIG. 7.**
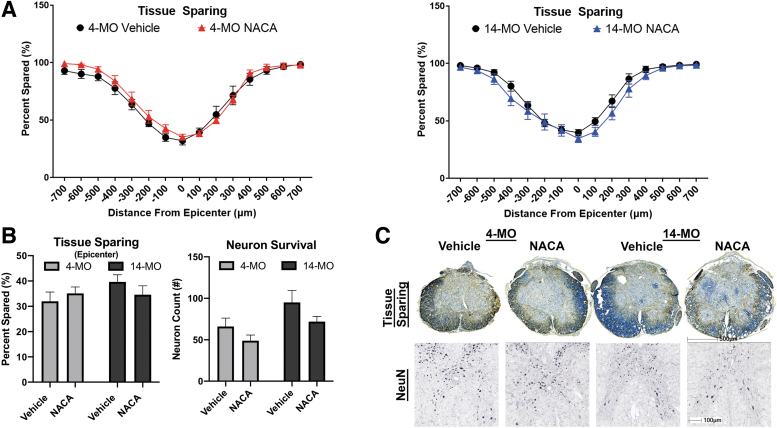
Treatment with N-acetylcysteine amide (NACA) does not affect tissue sparing nor neuron survival in 4- or 14-month-old (MO) mice after spinal cord injury (SCI). (**A–C**) NACA (150 mg/kg/day) was given to 4- and 14-MO mice after SCI, and tissue sparing and neuron survival around the lesion epicenter was assessed at 28 days post-injury (dpi). No significant effects were observed for any group on any outcome. (**A**) Tissue sparing was assessed throughout the lesion, and spared tissue was normalized to total tissue area. (**B,C**) Effects of NACA on sparing at the lesion epicenter were analyzed separately. (**B,C**) Neuron counts from ventral horns on sections from every 100 μm up to 500 μm rostral and caudal to the lesion epicenter were quantified and summed for analysis. (**C**) Images were stained with eriochrome cyanine and neurofilament 200 kD to assess tissue sparing. Images for tissue sparing were representative of group means at the lesion epicenter. Images for neuronal nuclear protein (NeuN) counts were representative of 500 μm rostral to the lesion epicenter and are representative of group means. (A) Assessments were performed using two-way repeated measures analysis of variance (ANOVA) for each age separately. (B) Assessments were performed using two-way ANOVA. *n* = 8–10/group. Graphs represent mean ± standard error of the mean. Scale bars = 500 μm for tissue sparing and μm for NeuN stained sections.

At 28 dpi, spinal cords were collected for anatomical analyses. Consistent with functional outcomes, no effects of NACA were observed in tissue outcomes. NACA had no effects on tissue sparing throughout the lesion in 4- (F_(1, 15)_ = 0.94, *p* = 0.34) or 14-MO mice (F_(1, 17)_ = 2.50, *p* = 0.13; Fig. 7A) or at the lesion epicenter (F_(1, 32)_ = 0.08, *p* = 0.76; Fig. 7B,C). A main effect of treatment with NACA also trended toward toxic effects on neuron survival surrounding the lesion independent of age (F_(1, 33)_ = 3.74, *p* = 0.06; Fig. 7B,C). Interestingly, however, and consistent with our previously published results,^11^ 14-MO mice had more neurons within 1 mm surrounding the lesion compared with 4-MO mice as a main effect of age (F_(1, 33)_ = 6.25, *p* < 0.01).

## Discussion

The main novel findings from this study are that (1) increased age is associated with lower levels of spinal GSH and an elevation of antioxidant response pathways consistent with a pro-oxidative environment in the absence of SCI. (2) SCI induces an early antioxidant response indicated by increased GPx activity by one dpi and diminishes the antioxidant substrate GSH by three dpi in 4- but not 14-MO mice. (3) Protecting GSH from depletion with age and SCI using cysteine supplementation provides evidence that the rate limiting component to GSH production is substrate in nature, rather than enzymatic, in SCI models.

Including age as a biological variable is understudied in the SCI field and is not commonly considered in clinical trials testing therapeutics.^39^ We and others have previously demonstrated that the biological response to SCI changes with age.^5,7,10,11,40–43^ Further, corresponding treatment effects targeting these adaptations do not always behave in predictable ways.^11^ For example, we have identified that using the uncoupler 2,4-dinitrophenol (DNP) to mitigate injury-induced mitochondrial ROS production exerts reciprocal effects on 4- and 14-MO mice, specifically eliciting toxicity to 4-MO-, and a therapeutic effect to 14-MO mice after SCI.^11^

While the effects of treatment on behavior and tissue pathology did not reach statistical significance in this study, trends across all outcomes allude to yet another possible age-specific drug interaction, being that older mice may have toxic effects to NACA treatment. This outcome is unexpected considering the cumulative work described throughout this study that identified older mice having less GSH, as well as previous findings of increased ROS production with age.^5,41^

We observed that spinal levels of GSH were consistently lower in older sham-injured mice. This suggests that older mice have a pre-existing depletion of spinal GSH that may approach a floor effect, prohibiting injury-induced compensations in antioxidant defenses. Indeed, we did not observe an injury effect in 14-MO mice across several outcomes including total and free-GSH, 3-NT accumulation, or GPx activity. Older mice did, however, have higher levels of GPx and GR activity independent of SCI. An inability to further increase antioxidant defenses after injury could sensitize older spinal cords to changes in oxidative stress.

While injury did not appear to further exacerbate oxidative stress by three dpi in older mice (Fig. 2), we have previously reported that both 3-NT and 4-HNE accumulate significantly more in 14- compared with 4-MO mice by seven dpi.^5,11^ Not finding an age-dependent increase in oxidative stress by three dpi in this study is consistent with our previous reports that also did not detect an increase in ROS accumulation by three dpi.^5^

Between three and seven dpi marks the emergence of macrophage infiltration within the spinal cord,^44^ whereby we have identified significant age-dependent increases in ROS production by macrophages.^7^ Observing a significant age-dependent increase in oxidative stress at seven dpi in our previous reports, but not three dpi in our previous report as well as in this study, argues that macrophages are likely a key player in the age-dependent increase in ROS damage that emerges after the three dpi time point assessed in this work.

In addition, identifying a significant decrease in GSH at three dpi, but not one dpi, further suggests that the emergence of macrophages at three dpi, and their production of ROS, may play a key role in depleting antioxidant reserves after SCI. Indeed, Nishi and colleagues (2020)^45^ have recently determined that aging-induced deficits are mitigated in immunocompromised Rag2Gamma^-/-^ mice, supporting the role of inflammation on age-dependent pathogenesis after SCI.

A potentially appropriate interpretation from our work, and one that goes against the original hypothesis is that the age-dependent changes in GSH are not essential to mediating age-dependent differences in oxidative stress from sources occurring in the acute and early subacute stages of SCI. Taken together, these findings help to refine our understanding of what biological underpinnings are changing with age that exacerbate the SCI pathophysiology and implicates pathophysiological responses occurring between three and seven dpi as critical to an age-dependent response.

Because cellular levels of GSH are considered the most abundant, and potentially the most important, source of antioxidant defense, much debate has been made as to what limits GSH production in pathological situations.^19,20^ During GSH synthesis, the ligation of glutamate and cysteine by GCLC is agreed to be the rate-limiting step to production.^46^ Whether GSH production is restricted by substrate or enzyme availability is debated.^19,20^

Our data argue that the limitation to GSH production after SCI is substrate availability. First, GCLC expression remains stable with age and SCI yet GSH is decreased, suggesting that either there is not enough substrate to saturate available enzyme, or GSH is being used faster than it can be produced. Providing NACA as a cysteine supplement significantly increased GSH production after SCI, demonstrating that increased availability of substrate will necessitate more production of GSH under these conditions. Based on these findings, it is likely that cysteine is a substrate limitation to GSH production with age and SCI.^47^

How cysteine availability is affected by SCI remains unknown, but previous reports have found that increased age is associated with both a total decrease in plasma cysteine as well as more cysteine existing in its oxidized form, cystine, which may be less accessible for GSH production.^47^ A decrease in available cysteine with advancing age could partially explain why GSH levels are lower within the spinal cord of our older sham-injured mice.

Because our data allude to availability of cysteine being the rate-limiting substrate to GSH maintenance after SCI, we sought to determine whether treatment using NACA as a cysteine analog could increase GSH and protect against oxidative stress after injury. Previous studies in rats using NAC or NACA to manage SCI have found beneficial therapeutic effects.^8,9,27,28,48,49^ In this study, NACA was first given to naïve mice to reproduce a previously published dosing strategy that increases cellular GSH to demonstrate its potential for augmenting GSH within the spinal cord. After demonstrating the efficacy of our approach in naïve mice, both 4- and 14-MO mice received SCI and were treated with NACA, which did augment spinal GSH and improved the GSH/GSSG redox ratio. These findings are consistent with previous literature utilizing NACA to treat SCI in rats^9^; however, in contrast to rat models, long-term outcomes of NACA treatment did not result in improved outcomes in 4-MO mice and trended toward toxicity in 14-MO mice.

Interestingly, Gou and coworkers (2015)^50^ utilized NAC, NACA's predecessor, to treat SCI in mice and also reported toxic effects on the BMS (Fig. 5A in referenced paper) with exacerbated white matter loss (Fig. 3B in referenced paper) within the lesion. Therefore, it is important to interpret our results in light of potential species differences that may mediate toxic effects to this compound as opposed to beneficial effects that have been reported in both rats and humans.^9,25,31,32^

Alternative mechanisms may also underlie our observation of no protective effects in younger mice, or trends toward toxicity in older mice. Specifically, cysteine has been shown to act as a natural anticoagulant that could exacerbate bleeding into the spinal cord.^51–53^ Further, high levels of cysteine have been found to drive the Fenton reaction that produces superoxide in the presence of iron, which could exacerbate oxidative stress. Finally, past literature has suggested that cysteine can act as an allosteric modulator to NMDA receptors and mediate increased excitotoxicity,^55–57^ of which excitotoxicity is a well-established contribution to secondary injury after SCI.

It is possible for any, or all, of these mechanisms to have occurred after NACA delivery and competed with potential beneficial effects derived from increasing cellular GSH. It is, therefore, essential for future work to identify the most appropriate dosing strategy to mitigate potential adverse effects of cysteine supplementation. It could be possible that our strategy, which was adopted from rat literature,^9^ was not optimal for mice. Specifically, our NACA dosing strategy could have been delivered at either too high a concentration for mice or too soon after SCI, leading to increased toxic mechanisms described above.

In this study, we observed older mice having slightly higher mean scores in the horizontal ladder, tissue sparing, and neuron counts surrounding the lesion, with no observable differences on the BMS compared with younger mice. This is in contrast to numerous previous observations of age-dependent decreases in functional and anatomic outcomes after SCI. It is difficult to know why we did not observe worse functional and histological outcomes with older age in this study. Several of our previous reports have found robust differences in outcomes between these ages.^5,7,10,11^ Several other reports have corroborated our assertations that older animals perform worse after SCI.^41,58–60^

One possible explanation for observations made in this study come back to the possibility of cohort-to-cohort variability. This is a limitation with including age as a biological variable that cannot be overcome. It is impossible to test the effects of different ages on SCI using the same litters at the same time. This limitation will always be important for interpretation when comparing two different ages performed in the same experiment. Observing an age-dependent effect of injury and pathology across multiple studies, laboratories, and throughout time is the most robust predictor of reproducibility.

In light of our study not demonstrating an age effect in behavior and tissue outcomes, however, another important consideration worth further investigation is the possibility that different cohorts of mice could age differently. Our observation of increased neurons surrounding the lesion in older mice is consistent with our previous report even in cohorts of mice that displayed an age-dependent reduction in function.^11^ Unfortunately, we do not know how to explain more neurons surrounding the lesion with advanced age.

There are other findings in this work that remain unexplained. Specifically, we observed an age-dependent increase in GSH peroxidase activity between sham-injured groups at one but not three dpi. There are several possible explanations for this outcome including cohort-to-cohort variability, differences in day-to-day protein preparation, or alternatively that sham-injured 4-MO mice had an acute decrease in GSH peroxidase activity that resolved by three dpi. In this study, we were interested in understanding the relationship between aging and SCI; therefore, we needed to include a control group of either naïve or sham. Because of feasibility considerations while running eight groups with equal representation in each experiment, we chose to include only sham and not naïve conditions. Although unlikely, it is important to interpret data with the potential for sham effects that could be observed within spinal homogenates, particularly early after surgery.

While sex was included as a biological variable in our studies, sex comparisons were not our primary end-point, and our studies were not powered to identify sex-specifics effects. We have provided a data table from all experiments utilizing both male and female mice in this article split by sex (Supplementary Table S1). The inclusion of both sexes across different ages led us to novel, incidental discoveries regarding secondary injury events after SCI.^66^ Sex differences in redox biology have been described across several systems,^61–63^ typically suggesting that female mice possess a greater capacity for antioxidant defense. While our data were underpowered to detect a sex-by-injury interaction in GSH availability, trends did exist (*p* = 0.068), with 4-MO male mice appearing to have the largest injury-induced decrease in GSH (Supplementary Table 1).

There are also a few caveats to consider regarding sex in our studies. Unlike humans, female mice do not experience menopause,^64^ and contrary to what is observed after reproductive senescence in humans, 14-MO female mice have higher levels of estradiol compared with 4-MO mice^11^. While the role of sex hormones on SCI has been interrogated in young rodents, little has been done to understand how changes in sex hormones with age affect SCI pathophysiology. Males and females do not age the same, so generalizing effects of aging between sexes should be interpreted with caution when only one sex is studied alone. It is therefore important to acknowledge that we did not include both sexes in our behavioral and histological NACA treatment experiment; therefore, future work would benefit from including sex as a biological variable when using antioxidants such as NACA to manage SCI.

It is important to note that seemingly modest effects observed throughout this study should be interpreted in light of tissue extraction methodology. Specifically, approximately 6.0 mm of tissue was isolated for molecular assessments, which is larger than the immediate lesioned environment that usually spans about 2.0–2.5 mm in length. Tissue responses to SCI will be different depending on whether they are within the immediate injured environment, within the lesion penumbra, or at distances far away from the injury.

Much of the effects tested are most logically associated within the lesion penumbra or immediate lesioned environment, and these regions might not comprise the largest percentage of protein obtained in tissue homogenates. What might appear to be a small magnitude of effect in total tissue homogenate could be a much larger magnitude of effect within the most relevant locations within the spinal cord. While diluting the lesion effect is not ideal, injured mouse spinal cord simply does not provide enough protein to run several molecular assessments when only the immediate lesioned environment is sampled. Detecting effects of injury, age, and treatment despite this possible limitation reinforces the strength of the associations found throughout this article.

## Conclusion

Collective work from this project has identified reduced antioxidant defense capacity with advanced age at time of SCI. Older age corresponded to a cellular profile consistent with a higher resting oxidative environment in sham-injured mice, and younger mice exhibited a similar oxidative response only after receiving a spinal contusion. This pre-existing increase in an oxidative response in older mice likely prohibited a further upregulation of antioxidant defenses. Data derived from this project suggest that the rate-limiting step to GSH synthesis after SCI is because of limited substrate availability, likely being available cysteine, which can be augmented by delivering NACA. Considering contrasting reports of beneficial effects of NACA in young rats after SCI, and the potential for both age- and species-specific effects, future work should investigate how age as a biological variable affects NACA treatment in larger animal models of neurotrauma.

## Supplementary Material

Supplemental data

Supplemental data

Supplemental data

## Data Sharing

All data from this manuscript will be publicly available through the Open Data Commons for Spinal Cord Injury. Images of original western blots are available in Supplementary File S1.
