# Supplementary material for: Advanced Age and Neurotrauma Diminish Glutathione and Impair Antioxidant Defense after Spinal Cord Injury
Source: J Neurotrauma. 2022 Jul 27;39(15-16):1075–89. doi: 10.1089/neu.2022.0010 (PMC9347421; doi:10.1089/neu.2022.0010)
Supplement: Supplemental data [file Supp_FigS1.docx]

**Supplementary Figure 1. Glutathione is diminished after SCI by 3-dpi in both 4- and 14-MO female mice, as well as between 4- and 14-MO sham-injured mice.** (**A**) Total, (**B**) Free/reduced, (**C**) Oxidized, (**D**) and the redox ratio (GSH/GSSG) were assessed in 4- and 14-MO mice with and without SCI at 1- and 3-dpi. (**A**) Total GSH was diminished by 3-dpi in both 4- and 14-MO mice as well as diminished in 14-MO sham-injured mice relative to 4-MO sham-injured mice. (**B**) Free/reduced GSH diminished by 3-dpi in both 4- and 14-MO mice. (**C**) Oxidized GSH was diminished by 3-dpi only in 4-MO mice and was diminished in sham-injured 14- compared to 4-MO mice. (**D**) No significant effects were observed in the redox ratio. Assessments were performed using two-way repeated measures ANOVA and used Sidak’s pair-wise comparisons as *post-hoc*. n=5/group. Graphs represent mean ± SEM. **p* < 0.05 relative to between age comparisons within day, ^†^*p* < 0.05 relative to sham-injured mice of the same age.


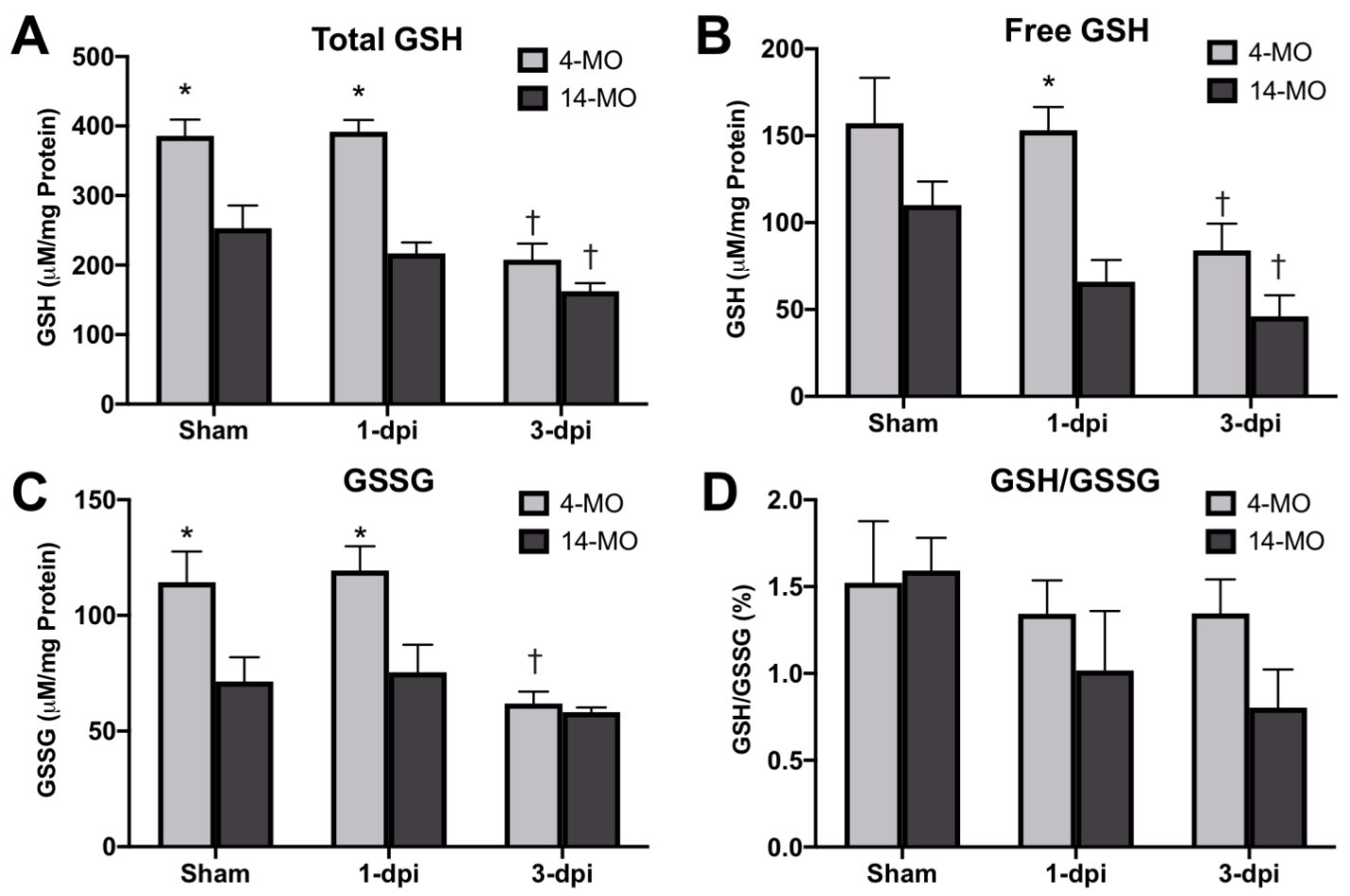


**Supplementary Table 1. Means, SD, and sample size of figures 2-4 split by sex, age, and injury.** Experiments utilizing both male and female mice were analyzed for sex by age, or sex by injury interactions using three-way ANOVAs to determine if further analyses should be performed on groups split by sex. When statistical interactions with sex were not identified, sexes were combined, and two-way ANOVAs were performed. Group means, standard deviations, and sample sizes are provided split by sex to enhance accessibility to our data prior to collapsing groups.
