# Supplementary material for: Advanced Age and Neurotrauma Diminish Glutathione and Impair Antioxidant Defense after Spinal Cord Injury
Source: J Neurotrauma. 2022 Jul 27;39(15-16):1075–89. doi: 10.1089/neu.2022.0010 (PMC9347421; doi:10.1089/neu.2022.0010)

1-DPI Western Blot Key

	Blot 1											
Animal ID	Liver	Ladder	9	6	14	11	1	3	19	16	35	32
Group ID	Control	Ladder	3	4	7	8	1	2	5	6	5	6
Injury	Liver	Ladder	SHAM	SCI	SHAM	SCI	SHAM	SCI	SHAM	SCI	SHAM	SCI
Age	Liver	Ladder	14-MO	14-MO	4-MO	4-MO	14-MO	14-MO	4-MO	4-MO	4-MO	4-MO
Gender	Liver	Ladder	F	F	F	F	M	M	M	M	M	M

	Blot 2											
Animal ID	Liver	Ladder	2	4	20	17	10	7	15	12	40	37
Group ID	Control	Ladder	1	2	5	6	3	4	7	8	7	8
Injury	Liver	Ladder	SHAM	SCI	SHAM	SCI	SHAM	SCI	SHAM	SCI	SHAM	SCI
Age	Liver	Ladder	14-MO	14-MO	4-MO	4-MO	14-MO	14-MO	4-MO	4-MO	4-MO	4-MO
Gender	Liver	Ladder	M	M	M	M	F	F	F	F	F	F

	Blot 3											
Animal ID	Liver	Ladder	28	8	23	5	38	13	33	18	30	27
Group ID	Control	Ladder	3	4	1	2	7	8	5	6	3	4
Injury	Liver	Ladder	SHAM	SCI	SHAM	SCI	SHAM	SCI	SHAM	SCI	SHAM	SCI
Age	Liver	Ladder	14-MO	14-MO	14-MO	14-MO	4-MO	4-MO	4-MO	4-MO	14-MO	14-MO
Gender	Liver	Ladder	F	F	M	M	F	F	M	M	F	F

	Blot 4											
Animal ID	Liver	Ladder	34	31	39	36	24	21	29	26	25	22
Group ID	Control	Ladder	5	6	7	8	1	2	3	4	1	2
Injury	Liver	Ladder	SHAM	SCI	SHAM	SCI	SHAM	SCI	SHAM	SCI	SHAM	SCI
Age	Liver	Ladder	4-MO	4-MO	4-MO	4-MO	14-MO	14-MO	14-MO	14-MO	14-MO	14-MO
Gender	Liver	Ladder	M	M	F	F	M	M	F	F	M	M

1-DPI GCLC

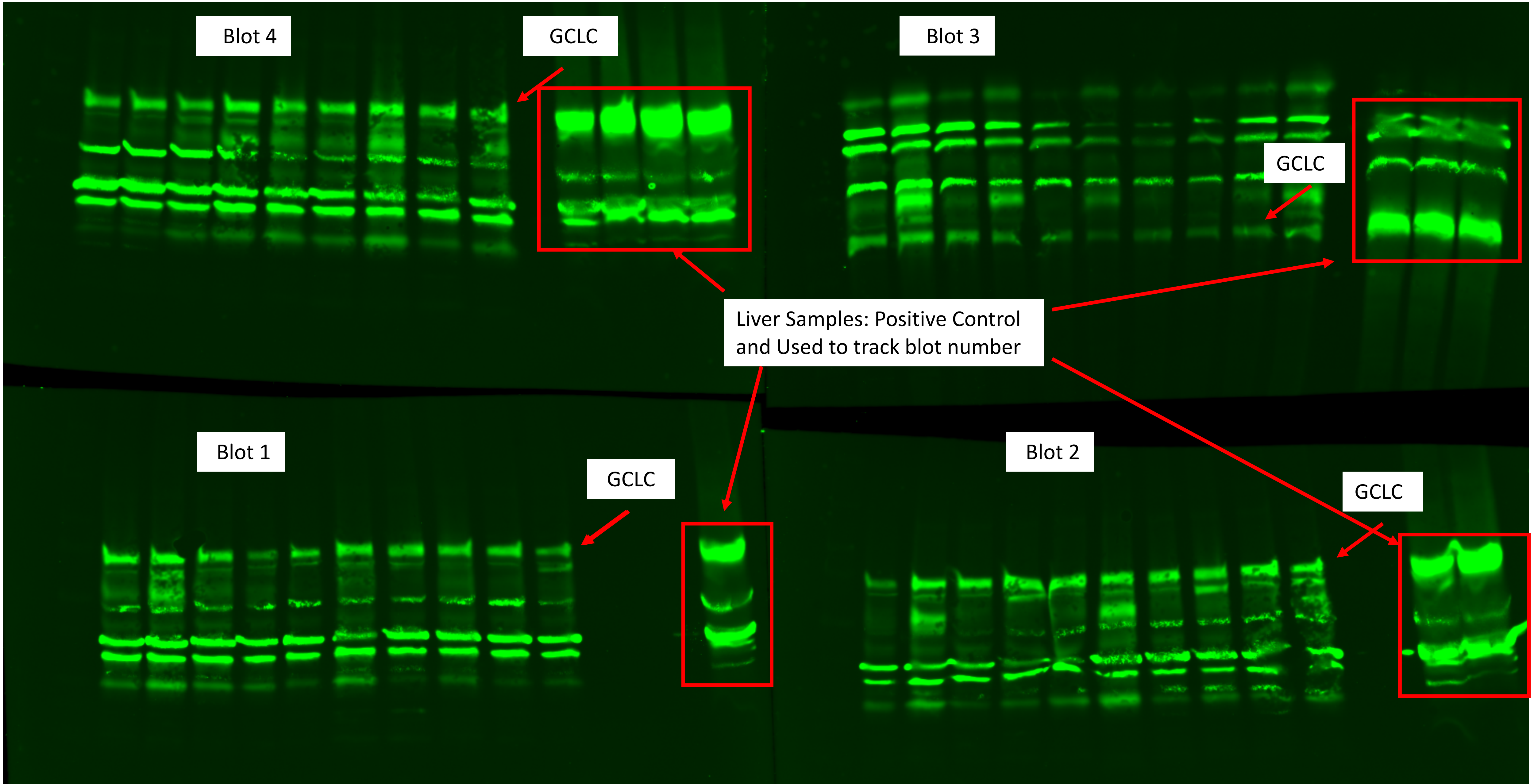
Blot 4
GCLC
Blot 3
GCLC
Liver Samples: Positive Control and Used to track blot number
Blot 1
GCLC
Blot 2
GCLC

1-DPI GapDH for GCLC

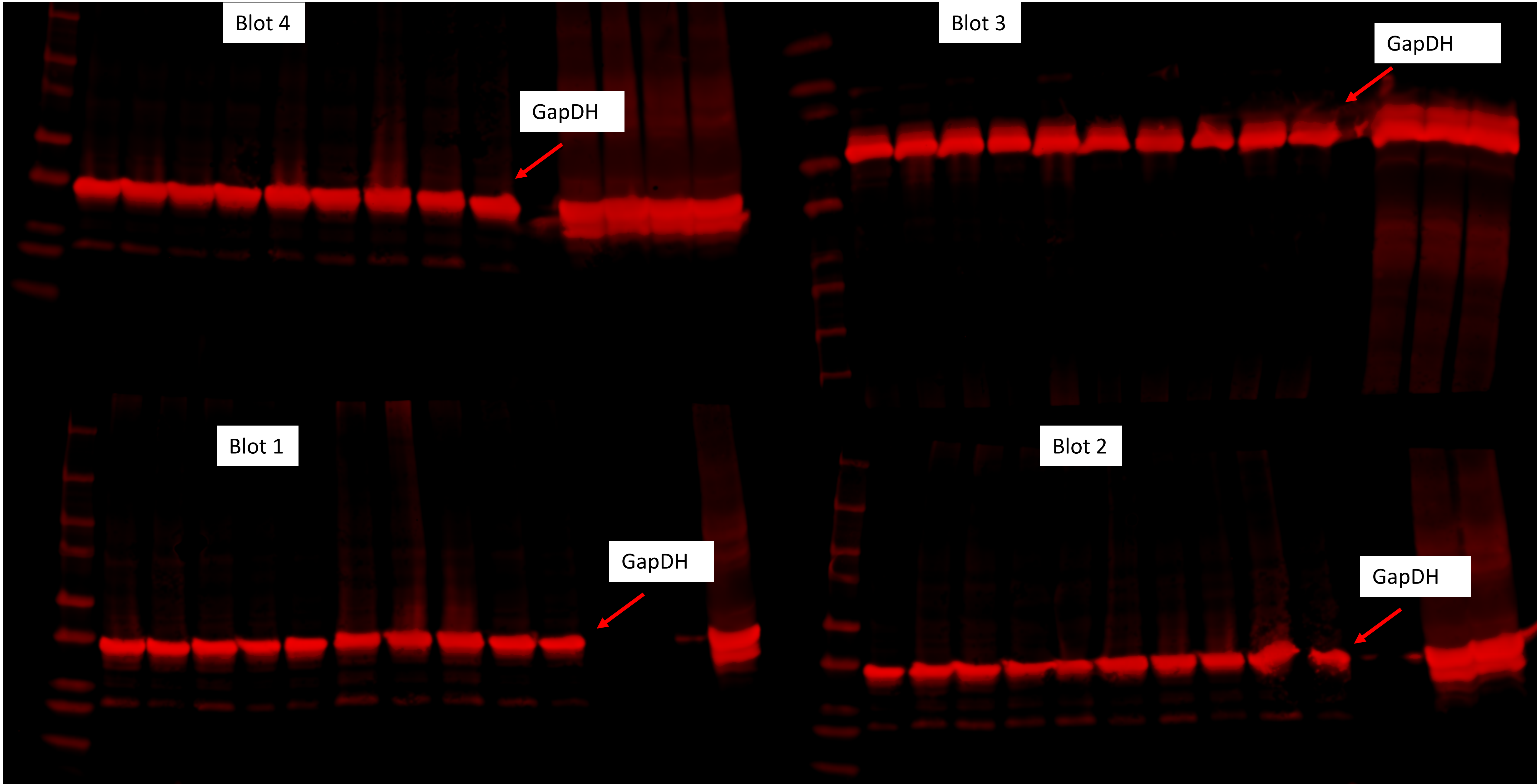

1-DPI GSH Reductase (Green)

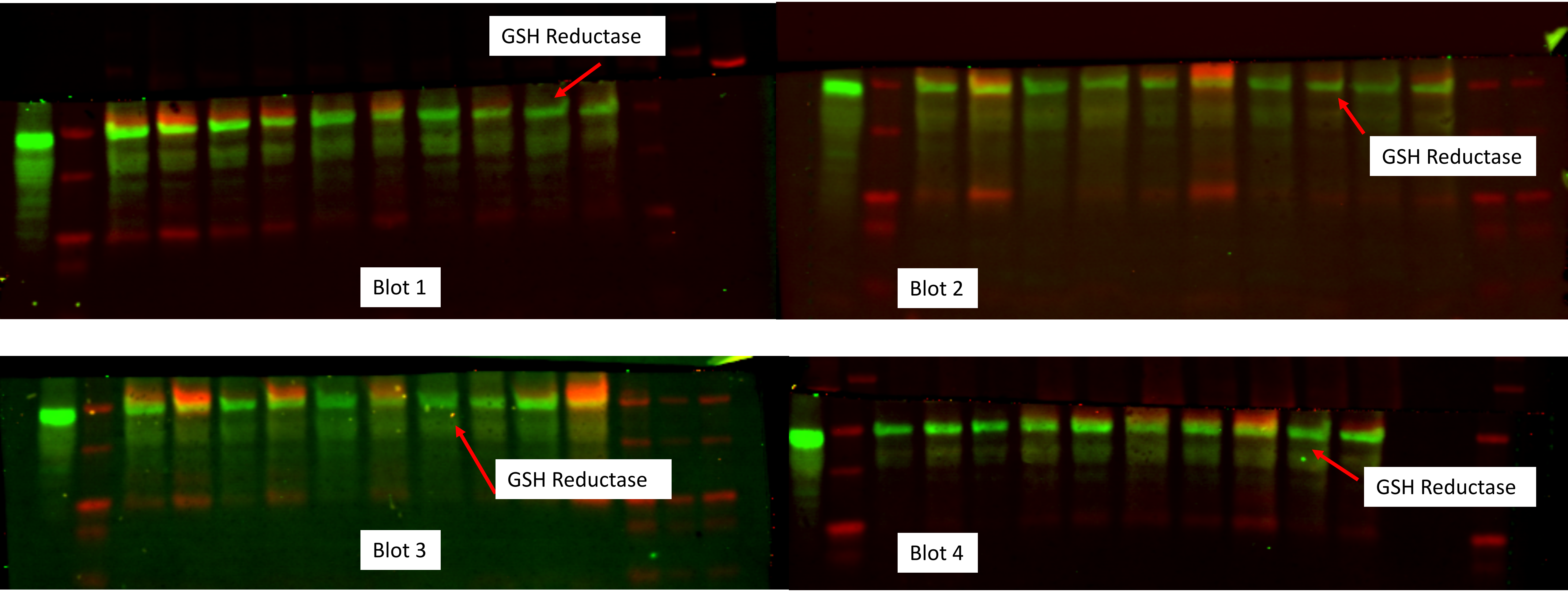

1-DPI GSH Synthetase (Red) and GapDH (Green)

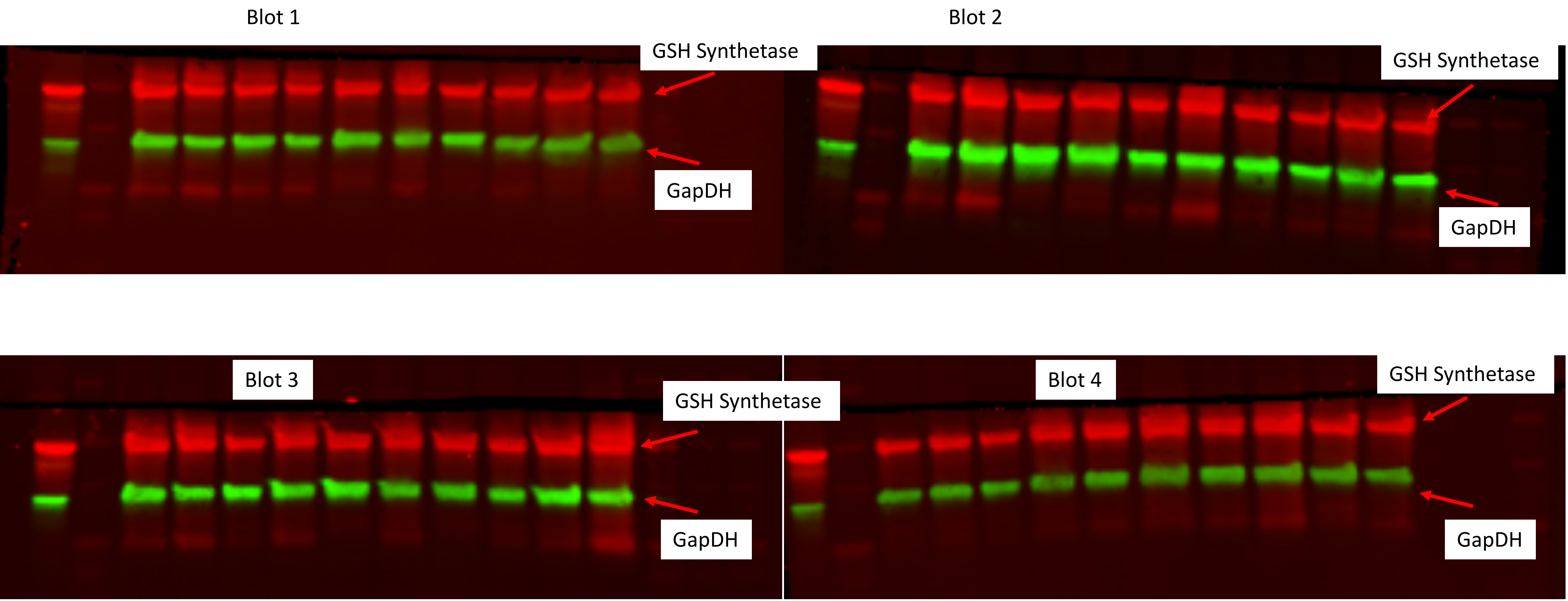

1-DPI GSH Peroxidase

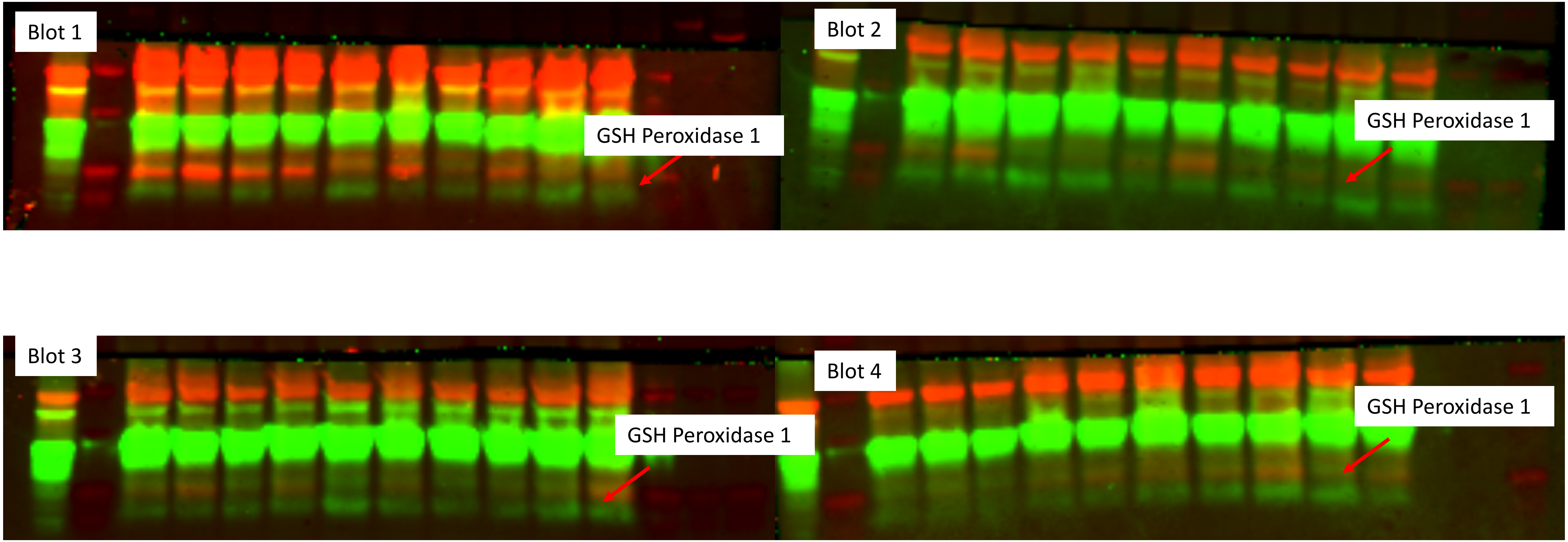

3-DPI Western Blot Key

	Blot 1										
Animal ID	Liver	Ladder	1	4	6	9	11	14	16	19	21	23
Group ID	Control	Ladder	4	3	8	7	2	1	6	5	4	3
Injury	Liver	Ladder	SCI	Sham	SCI	Sham	SCI	Sham	SCI	Sham	SCI	Sham
Age	Liver	Ladder	14	14	4	4	14	14	4	4	14	14
Gender	Liver	Ladder	F	F	F	F	M	M	M	M	F	F

	Blot 2											
Animal ID	Liver	Ladder	5	10	15	20	2	7	12	17	40	37
Group ID	Control	Ladder	3	7	1	5	4	8	2	6	5	6
Injury	Liver	Ladder	Sham	Sham	Sham	Sham	SCI	SCI	SCI	SCI	Sham	SCI
Age	Liver	Ladder	14	4	14	4	14	4	14	4	4	4
Gender	Liver	Ladder	F	F	M	M	F	F	M	M	M	M

	Blot 3											
Animal ID	Liver	Ladder	39	36	30	27	35	32	25	22	28	26
Group ID	Control	Ladder	5	6	7	8	1	2	3	4	7	8
Injury	Liver	Ladder	Sham	SCI	Sham	SCI	Sham	SCI	Sham	SCI	Sham	SCI
Age	Liver	Ladder	4	4	4	4	14	14	14	14	4	4
Gender	Liver	Ladder	M	M	F	F	M	M	F	F	F	F

	Blot 4											
Animal ID	Liver	Ladder	31	33	8	29	18	38	3	24	13	34
Group ID	Control	Ladder	2	1	8	7	6	5	4	3	2	1
Injury	Liver	Ladder	SCI	Sham	SCI	Sham	SCI	Sham	SCI	Sham	SCI	Sham
Age	Liver	Ladder	14	14	4	4	4	4	14	14	14	14
Gender	Liver	Ladder	M	M	F	F	M	M	F	F	M	M

3-DPI GCLC

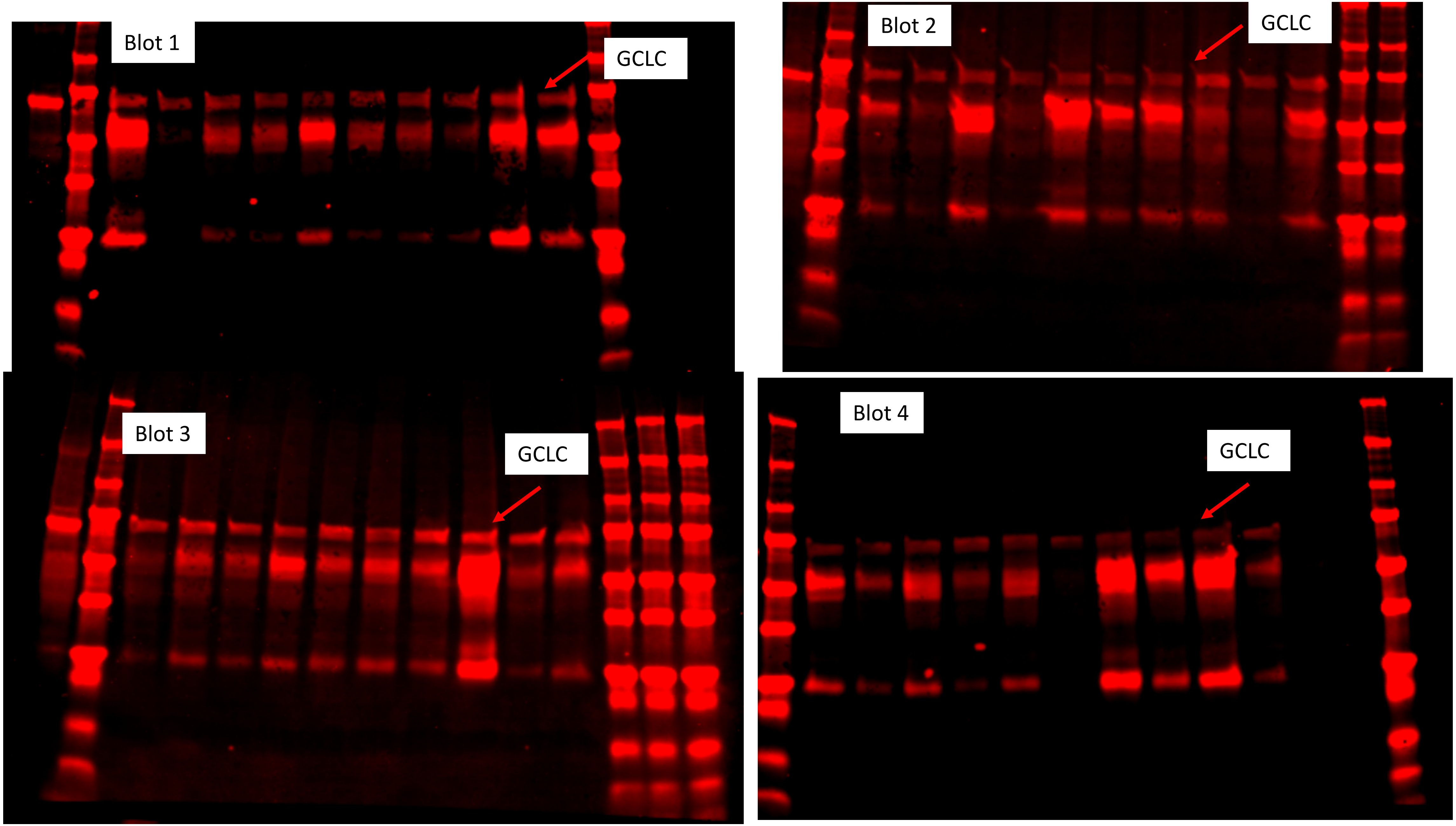

3-DPI GSH Synthetase

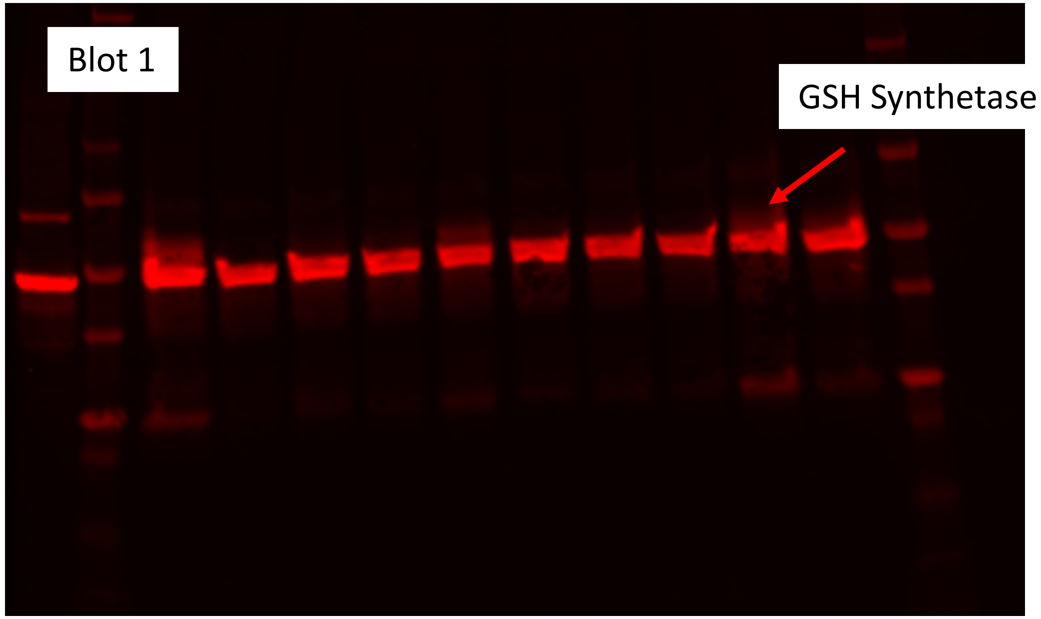

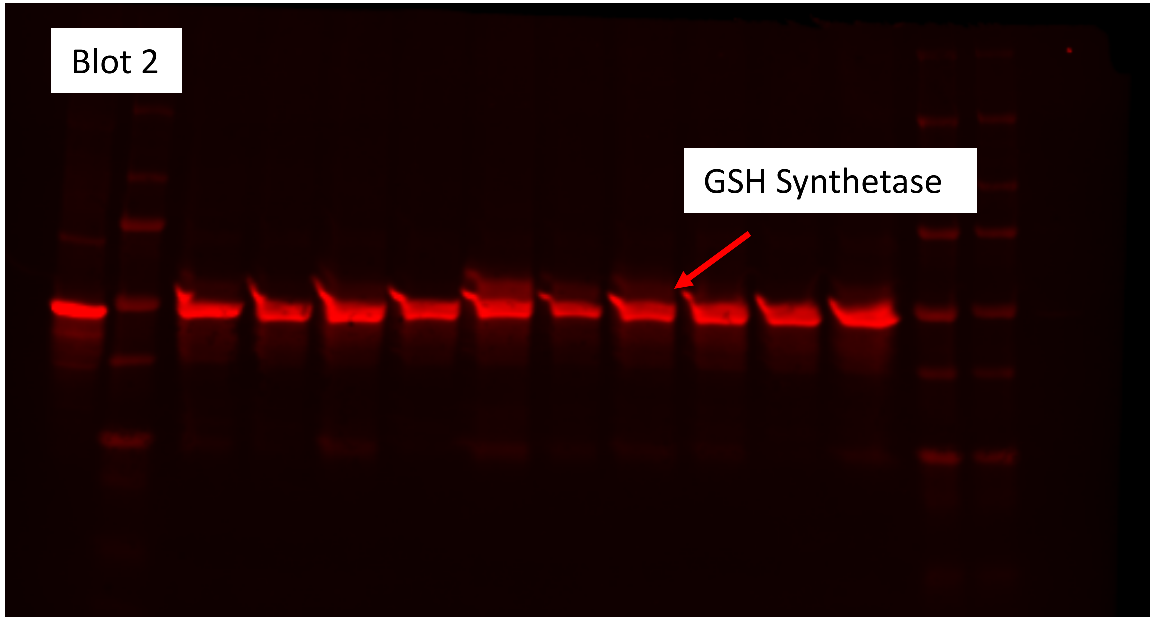

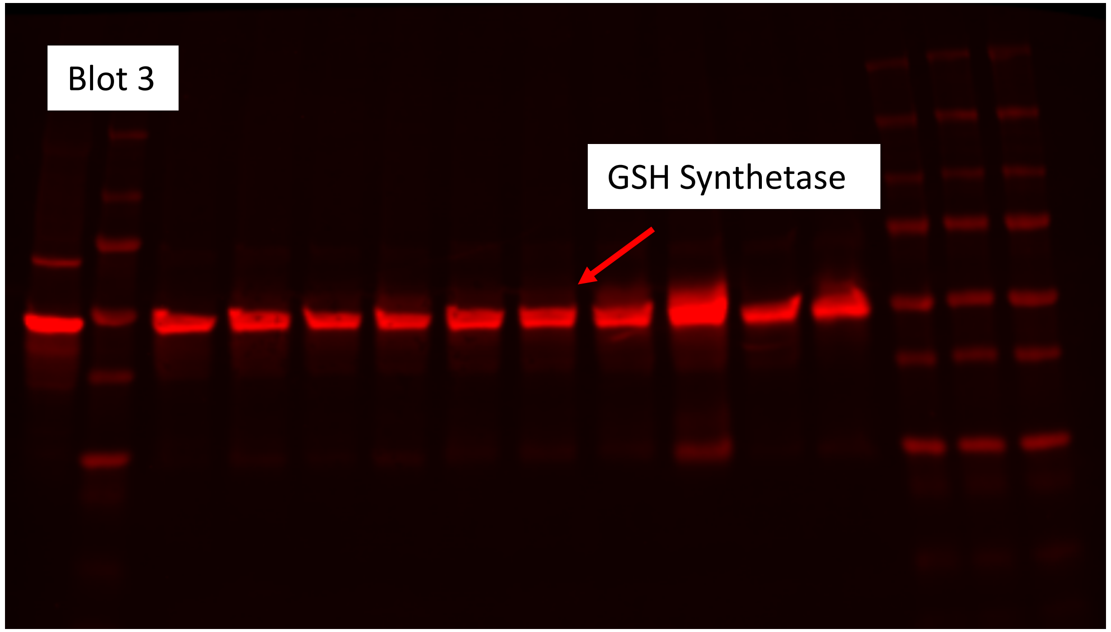

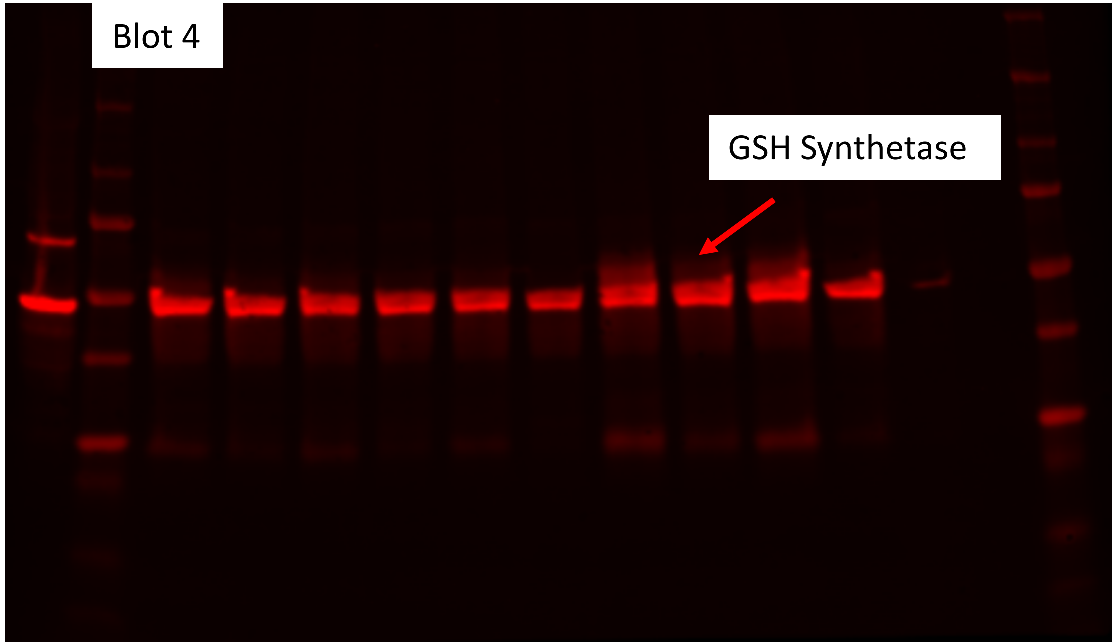

3-DPI GSH Reductase (Green)

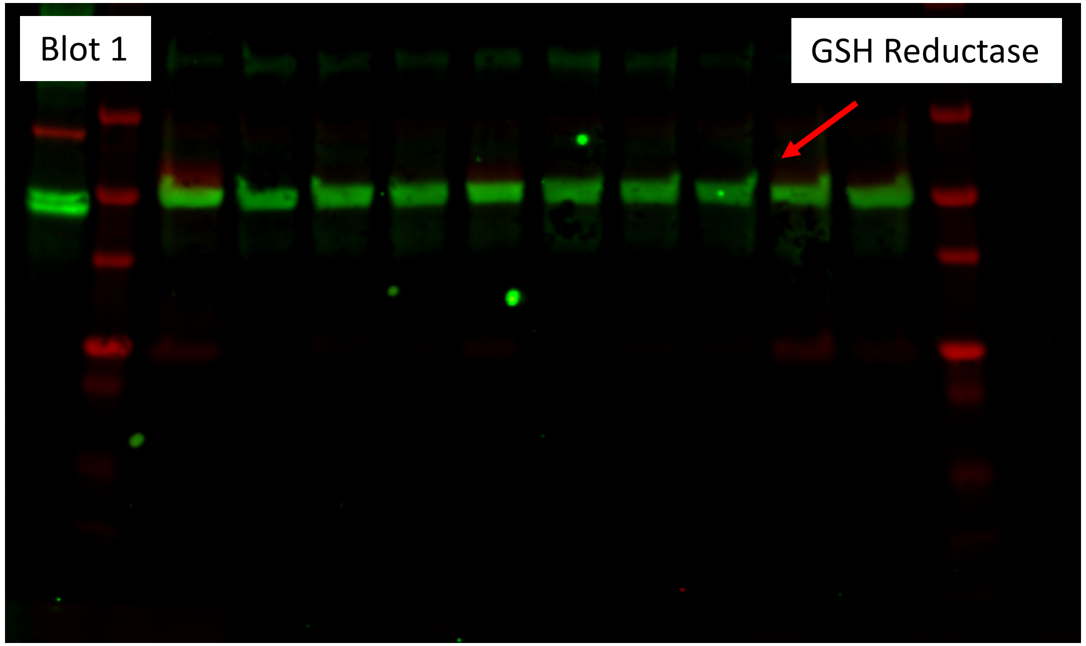

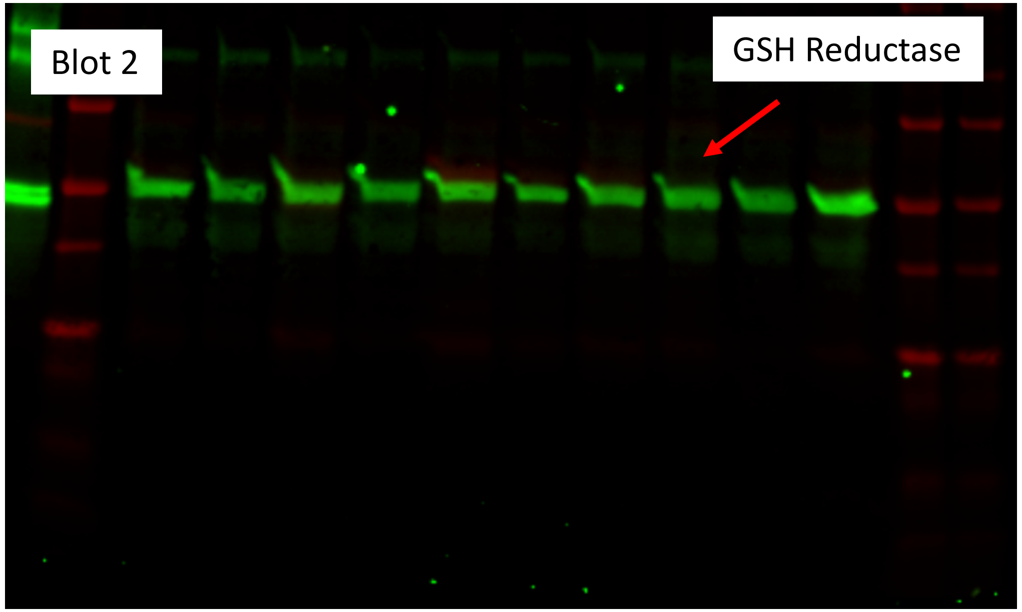

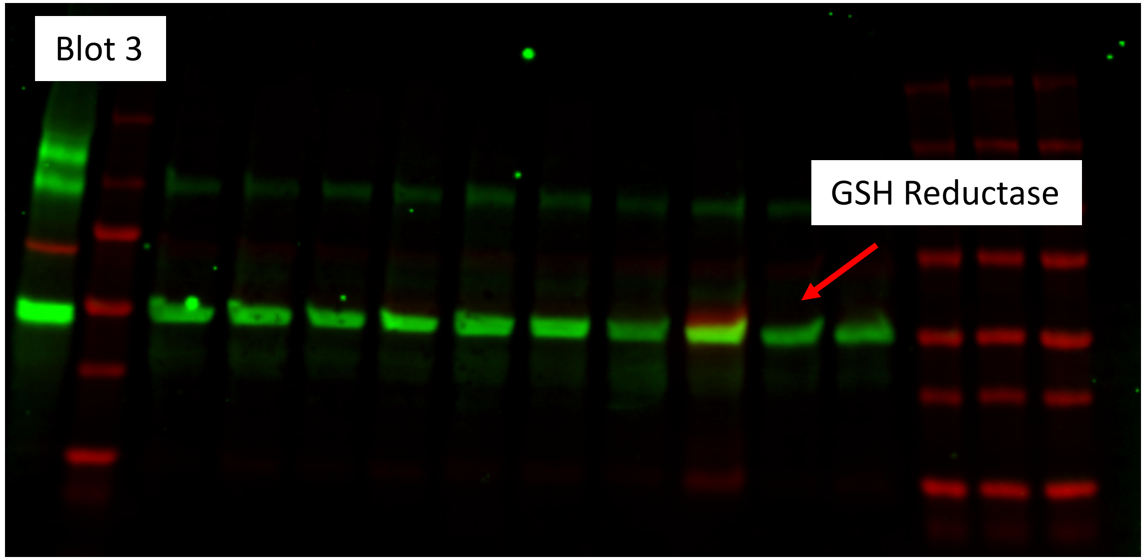

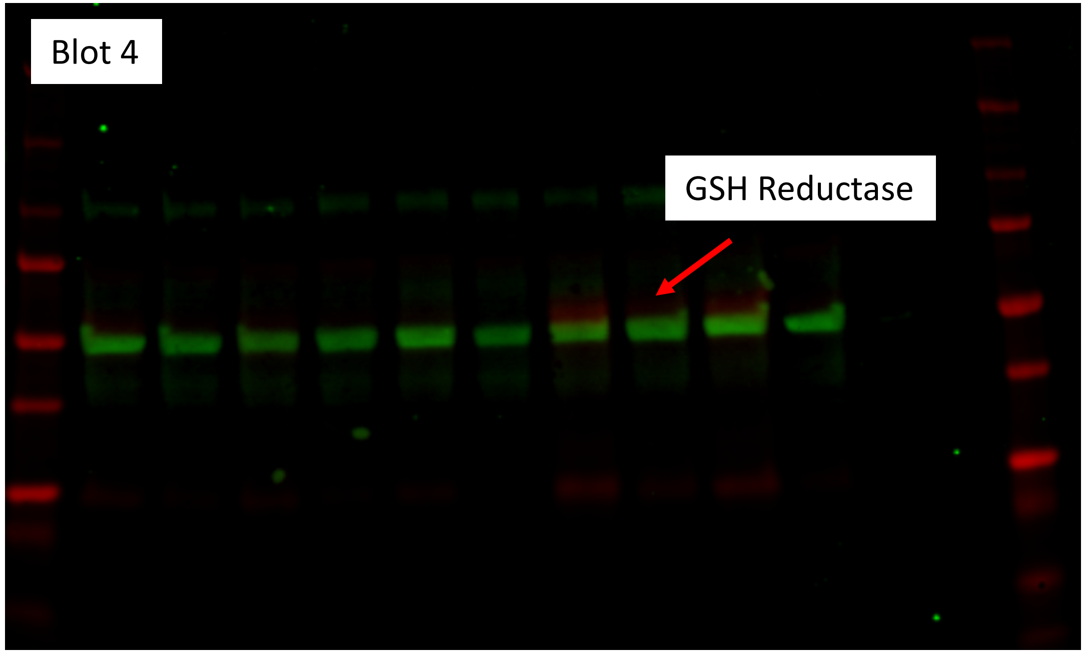

3-DPI GSH Peroxidase

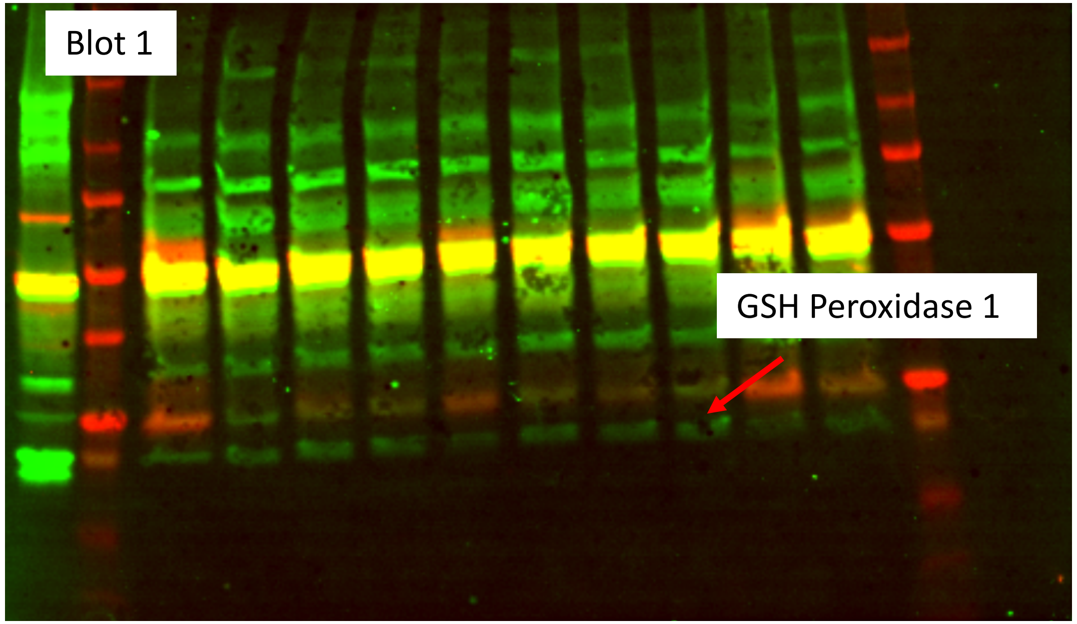

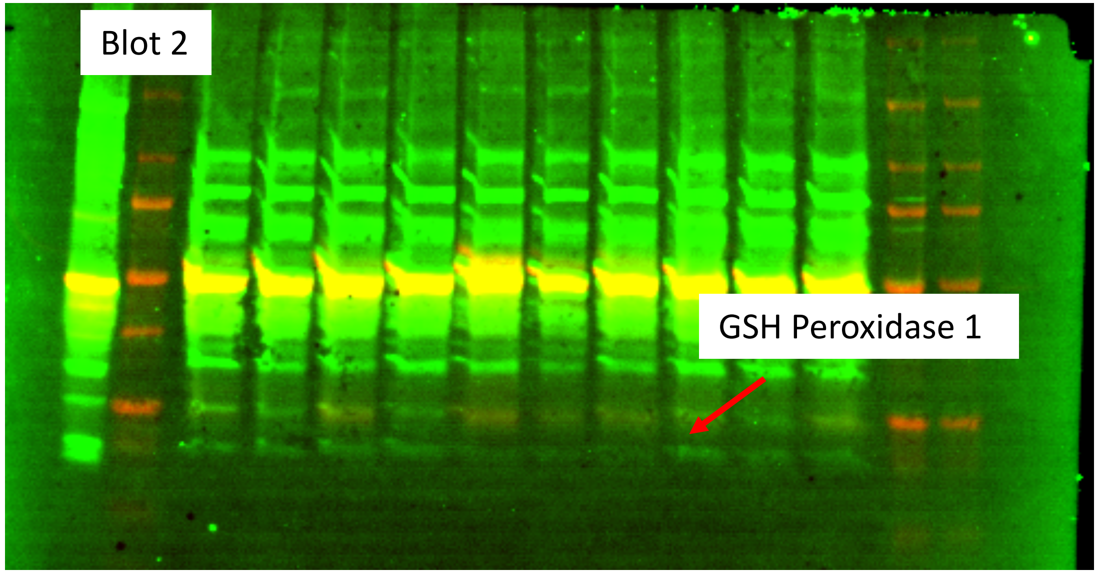

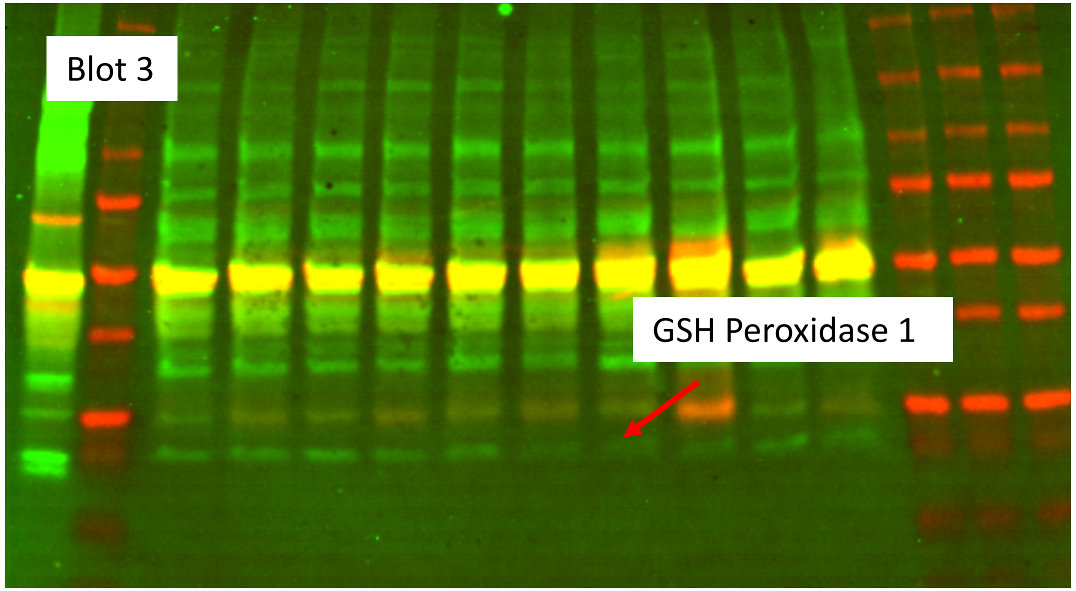

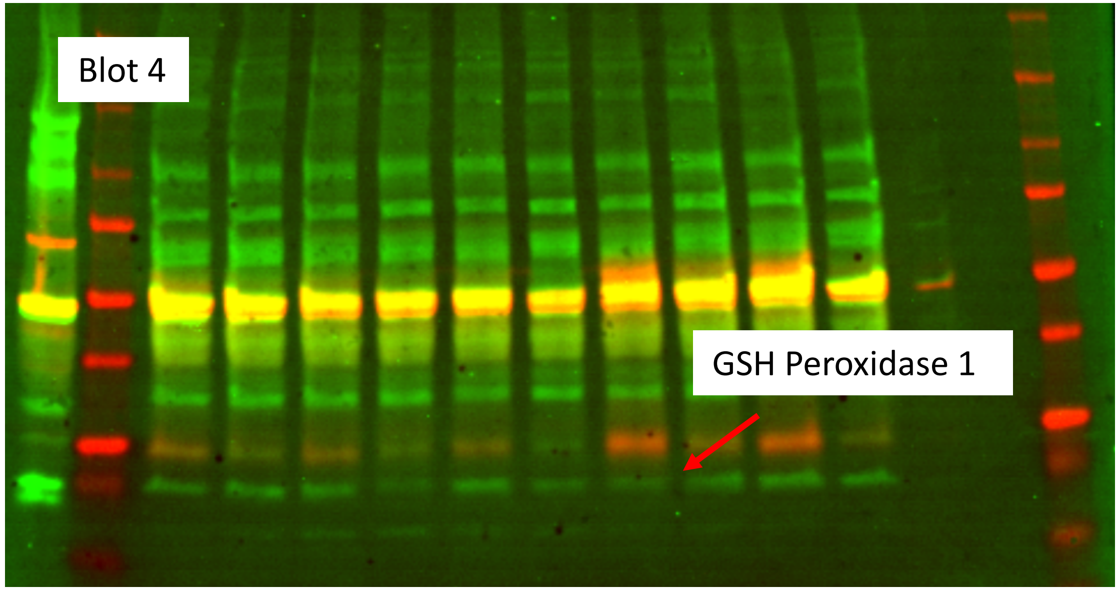

3-DPI GapDH (Green) GSH Synthetase (Red repeat from above)

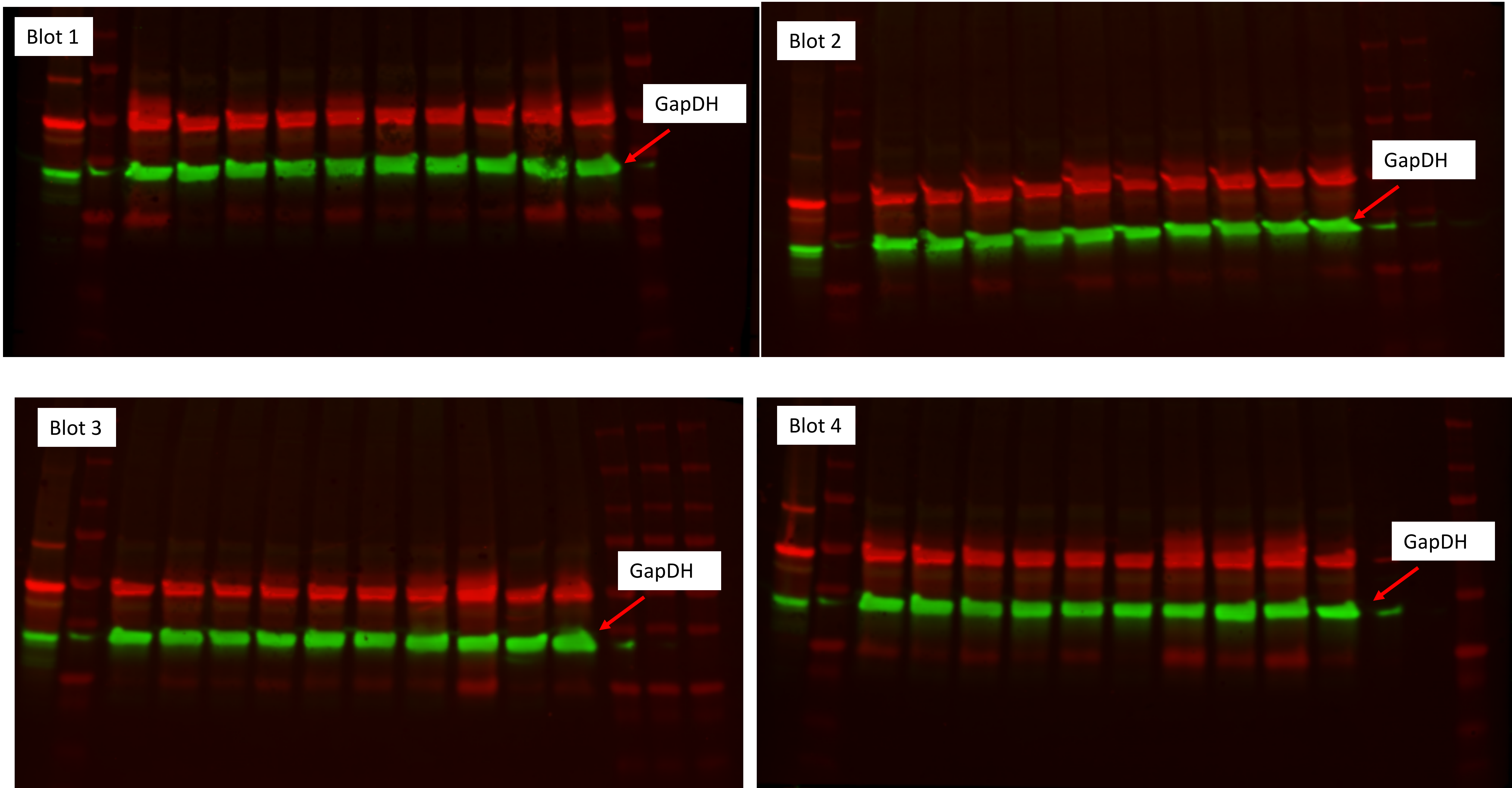

3-NT Dot Blot Key

Dots are placed in animal Order

Note No protein for animal 10 remained. Blot space was used as a negative control (solvent only)

Dot in bottom right is a positive control (BSA treated with peroxynitrite produced from SIN-1).

Unique ID	09172019-1	09172019-2	09172019-3	09172019-4	09172019-5	09172019-6	09172019-7	09172019-8	09172019-9	09172019-10
Animal ID	1	2	3	4	5	6	7	8	9	10
Group ID	4	4	4	3	3	8	8	8	7	7
Age	14	14	14	14	14	4	4	4	4	4
Sex	F	F	F	F	F	F	F	F	F	F
Injury	SCI	SCI	SCI	Sham	Sham	SCI	SCI	SCI	Sham	Sham

Unique ID	09172019-11	09172019-12	09172019-13	09172019-14	09172019-15	09172019-16	09172019-17	09172019-18	09172019-19	09172019-20
Animal ID	11	12	13	14	15	16	17	18	19	20
Group ID	2	2	2	1	1	6	6	6	5	5
Age	14	14	14	14	14	4	4	4	4	4
Sex	M	M	M	M	M	M	M	M	M	M
Injury	SCI	SCI	SCI	Sham	Sham	SCI	SCI	SCI	Sham	Sham

Unique ID	09172019-21	09172019-22	09172019-23	09172019-24	09172019-25	09172019-26	09172019-27	09172019-28	09172019-29	09172019-30
Animal ID	21	22	23	24	25	26	27	28	29	30
Group ID	4	4	3	3	3	8	8	7	7	7
Age	14	14	14	14	14	4	4	4	4	4
Sex	F	F	F	F	F	F	F	F	F	F
Injury	SCI	SCI	Sham	Sham	Sham	SCI	SCI	Sham	Sham	Sham

Unique ID	09172019-31	09172019-32	09172019-33	09172019-34	09172019-35	09172019-36	09172019-37	09172019-38	09172019-39	09172019-40
Animal ID	31	32	33	34	35	36	37	38	39	40
Group ID	2	2	1	1	1	6	6	5	5	5
Age	14	14	14	14	14	4	4	4	4	4
Sex	M	M	M	M	M	M	M	M	M	M
Injury	SCI	SCI	Sham	Sham	Sham	SCI	SCI	Sham	Sham	Sham

3-NT Dot Blot 3 DPI

Replicate 1

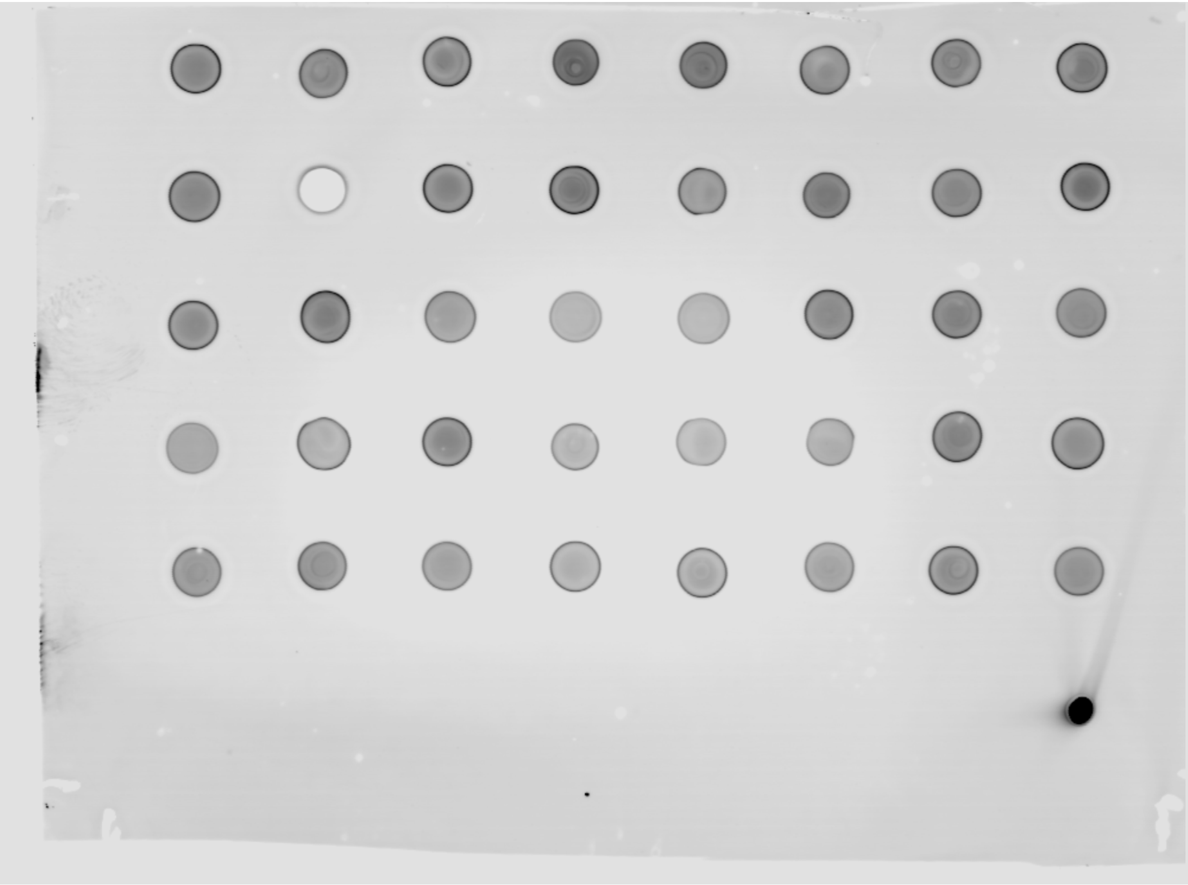

Replicate 2

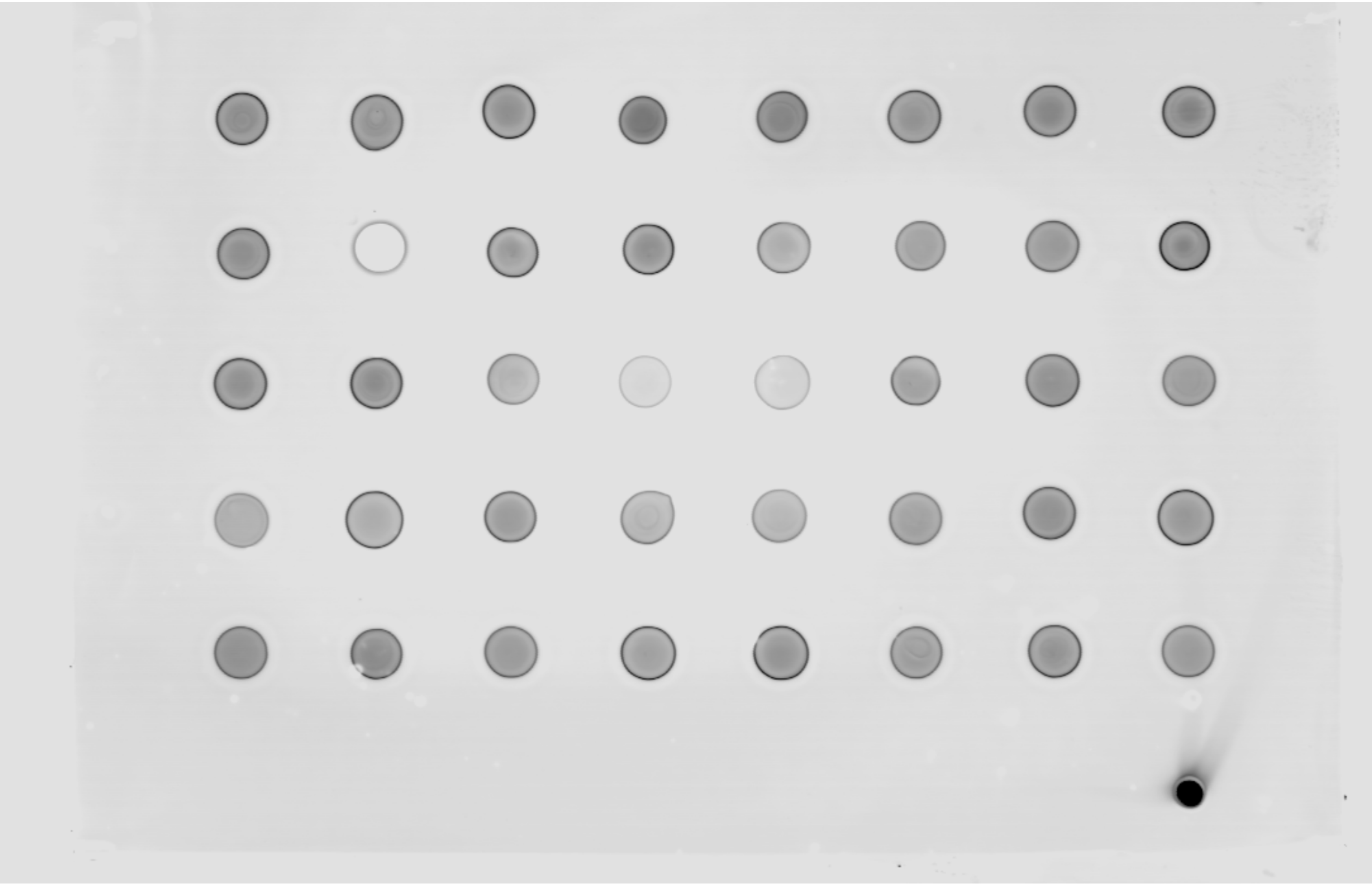

NACA Treated 3-NT Dot Blot Key 1-DPI

Dots are placed in animal Order

Animal ID	1	2	3	4	5	Blank	7	8	Blank	Blank
Group Code	1	2	1	2	1	Blank	1	2	Blank	Blank
Dose	Control	NACA	Control	NACA	Control	Blank	Control	NACA	Blank	Blank
Age	4	4	4	4	4	Blank	4	4	Blank	Blank
Sex	f	f	f	f	f	Blank	f	f	Blank	Blank

Animal ID	11	12	13	14	15	16	17	18	19	20
Group Code	1	2	1	2	1	2	0	2	0	0
Dose	Control	NACA	Control	NACA	Control	NACA	Naïve	NACA	Naïve	Naïve
Age	4	4	4	4	4	4	4	4	4	4
Sex	f	f	f	f	f	f	f	f	f	f

Animal ID	1	2	4	5	6	7	8	9	10
Group Code	1	2	2	1	2	1	2	1	2
Dose mg/kg	Control	NACA	NACA	Control	NACA	Control	NACA	Control	NACA
Age	14	14	14	14	14	14	14	14	14
Sex	f	f	f	f	f	f	f	f	f

Animal ID	11	12	14	15	16	17	18	19	20
Group Code	1	2	2	1	0	1	0	0	0
Dose mg/kg	Control	NACA	NACA	Control	Naïve	Control	Naïve	Naïve	Naïve
Age	14	14	14	14	14	14	14	14	14
Sex	f	f	f	f	f	f	f	f	f

3-NT Dot Blot NACA Treated 1-dpi

4-MO
Replicate 1

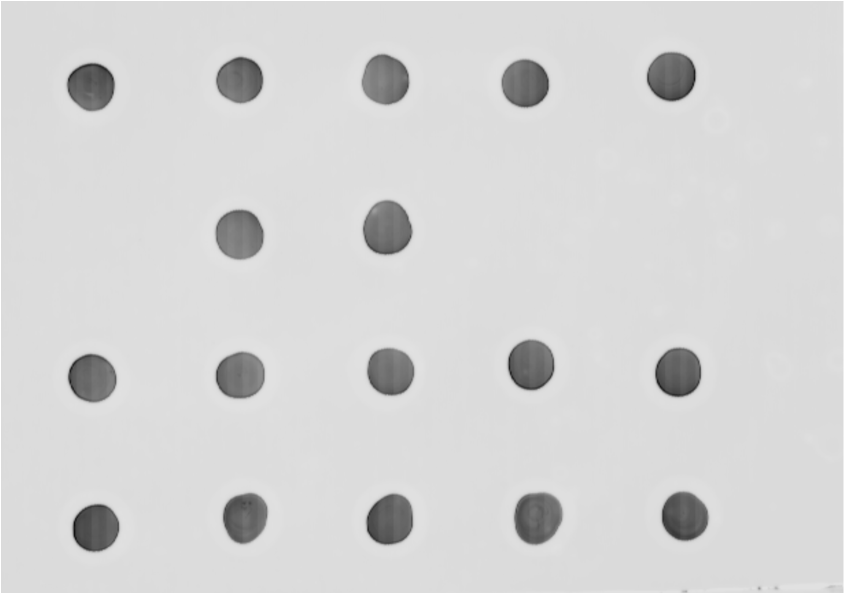

4-MO
Replicate 2

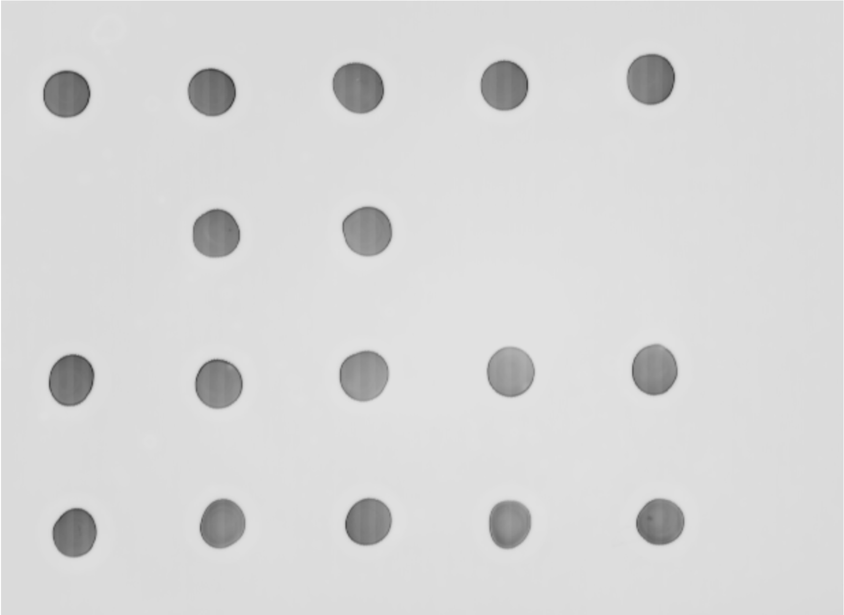

14-MO
Replicate 1

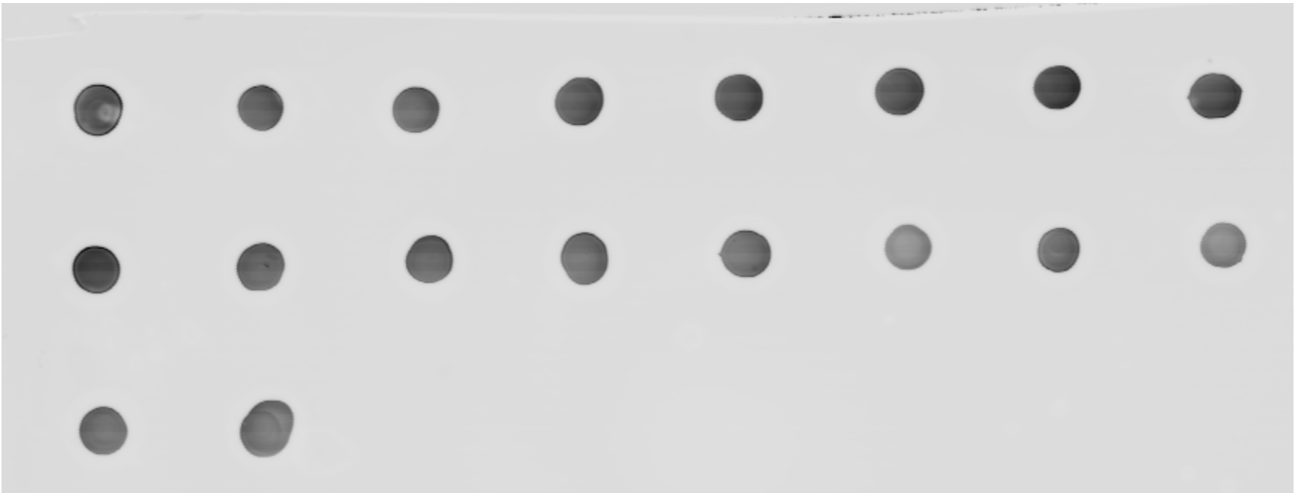

14-MO
Replicate 2

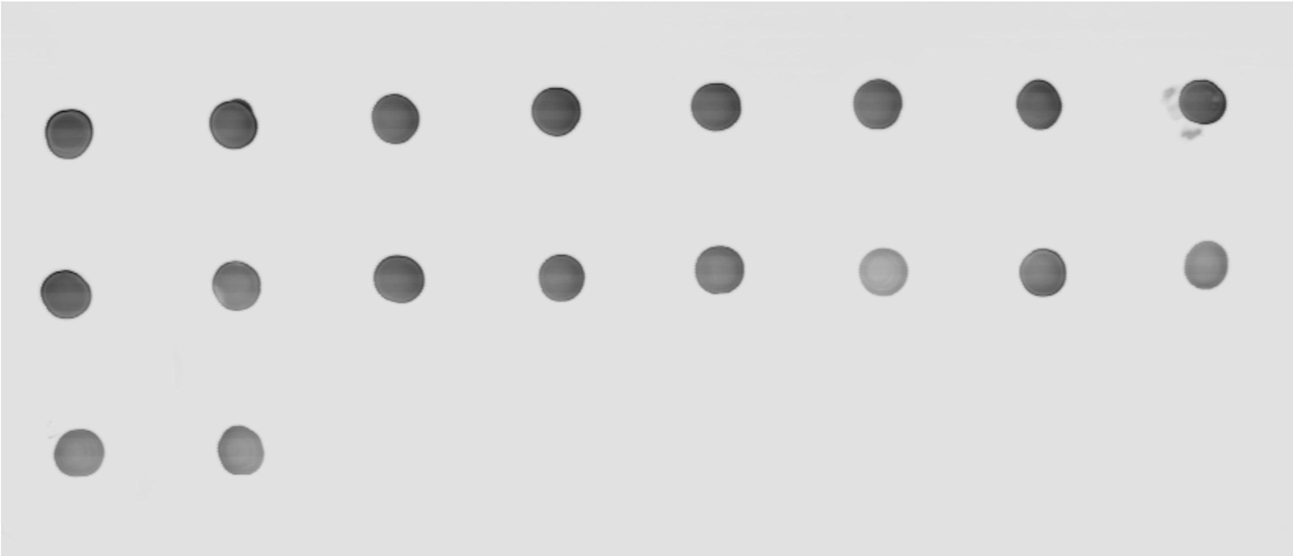

NACA Treated 4-HNE
Dot Blot Key 1-DPI

Dots are placed in
animal Order

Animal ID	1	2	3	4	5	Blank	7	8	Blank	Blank
Group Code	1	2	1	2	1	Blank	1	2	Blank	Blank
Dose	Control	NACA	Control	NACA	Control	Blank	Control	NACA	Blank	Blank
Age	4	4	4	4	4	Blank	4	4	Blank	Blank
Sex	f	f	f	f	f	Blank	f	f	Blank	Blank

Animal ID	11	12	13	14	15	16	17	18	19	20
Group Code	1	2	1	2	1	2	0	2	0	0
Dose	Control	NACA	Control	NACA	Control	NACA	Naïve	NACA	Naïve	Naïve
Age	4	4	4	4	4	4	4	4	4	4
Sex	f	f	f	f	f	f	f	f	f	f

Animal ID	1	2	Blank	4	5	6	7	8	9	10
Group Code	1	2	Blank	2	1	2	1	2	1	2
Dose mg/kg	Control	NACA	Blank	NACA	Control	NACA	Control	NACA	Control	NACA
Age	14	14	Blank	14	14	14	14	14	14	14
Sex	f	f	Blank	f	f	f	f	f	f	f

Animal ID	11	12	Blank	14	15	16	17	18	19	20
Group Code	1	2	Blank	2	1	0	1	0	0	0
Dose mg/kg	Control	NACA	Blank	NACA	Control	Naïve	Control	Naïve	Naïve	Naïve
Age	14	14	Blank	14	14	14	14	14	14	14
Sex	f	f	Blank	f	f	f	f	f	f	f

4-HNE Dot Blot NACA Treated 1-dpi

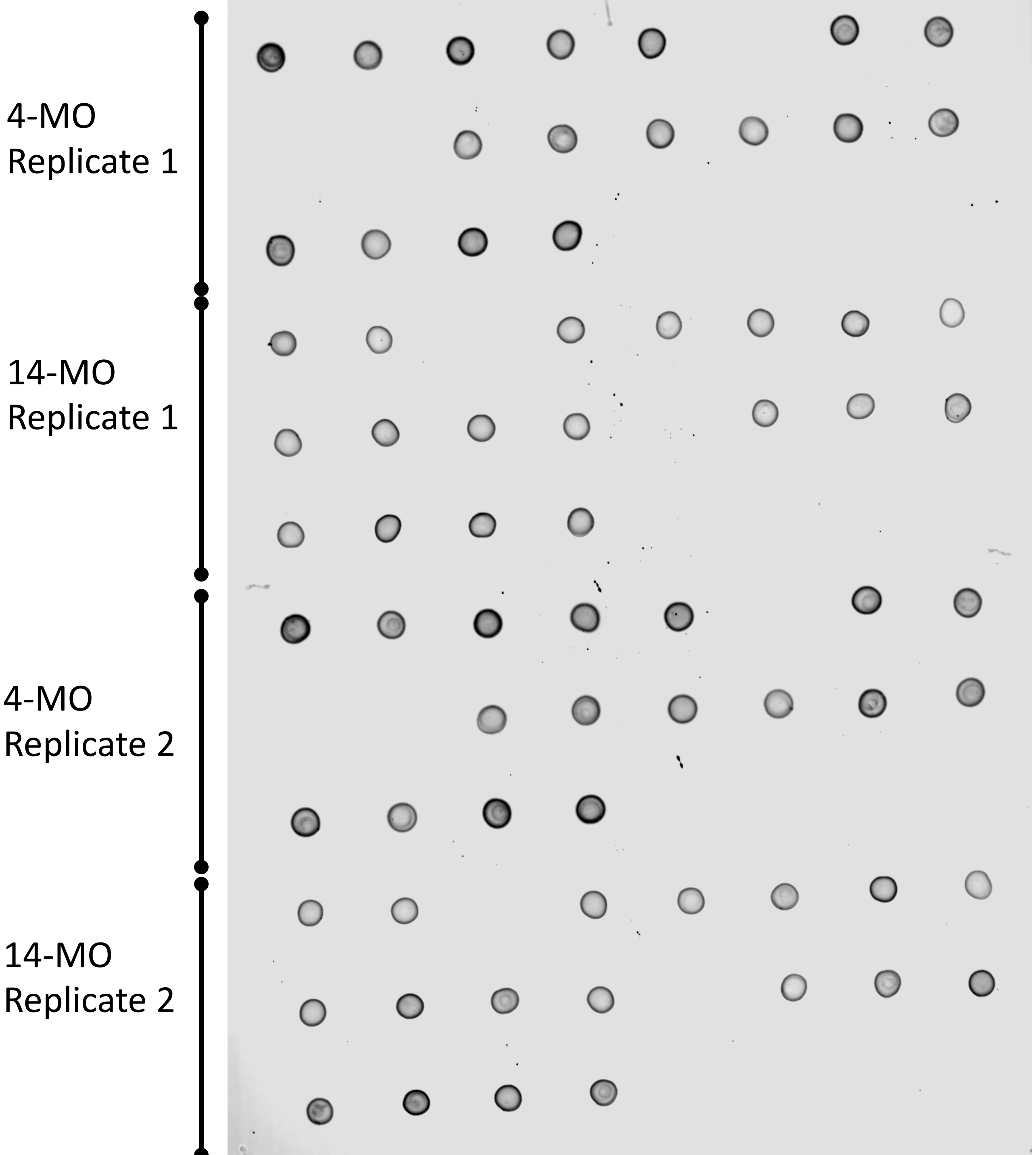

Supplement: Supplemental data [file Supp_Data.pdf]
